# Substrate-bound and soluble domains of tenascin-C regulate differentiation, proliferation and migration of neural stem and progenitor cells

**DOI:** 10.3389/fncel.2024.1357499

**Published:** 2024-02-14

**Authors:** Kristin Glotzbach, Andreas Faissner

**Affiliations:** Department of Cell Morphology and Molecular Neurobiology, Faculty of Biology and Biotechnology, Ruhr University Bochum, Bochum, Germany

**Keywords:** tenascin-C, alternatively spliced fibronectin III domains, neural stem/progenitor cells, differentiation, proliferation, migration, dichotomy of coated substrate and soluble additives

## Abstract

**Introduction:**

The lack of regenerative capacity of the central nervous system is one of the major challenges nowadays. The knowledge of guidance cues that trigger differentiation, proliferation, and migration of neural stem and progenitor cells is one key element in regenerative medicine. The extracellular matrix protein tenascin-C (Tnc) is a promising candidate to regulate cell fate due to its expression in the developing central nervous system and in the adult neural stem cell niches. Of special interest are the alternatively spliced fibronectin type III (FnIII) domains of Tnc whose combinatorial diversity could theoretically generate up to 64 isoforms in the mouse. A total of 27 isoforms have already been discovered in the developing brain, among others the domain combinations A1D, CD, and A124BCD.

**Methods:**

In the present study, these domains as well as the combination of the constitutively expressed FnIII domains 7 and 8 (78) were expressed in Chinese hamster ovary cells as pseudo-antibodies fused to the Fc-fragment of a human immunoglobulin G antibody. The fusion proteins were presented to primary mouse neural stem/progenitor cells (NSPCs) grown as neurospheres, either as coated culture substrates or as soluble additives *in vitro*. The influence of the domains on the differentiation, proliferation and migration of NSPCs was analyzed.

**Results:**

We observed that the domain combination A124BCD promoted the differentiation of neurons and oligodendrocytes, whereas the domain A1D supported astrocyte differentiation. The constitutively expressed domain 78 had a proliferation and migration stimulating impact. Moreover, most effects were seen only in one of the presentation modes but not in both, suggesting different effects of the Tnc domains in two- and three-dimensional cultures.

**Discussion:**

This knowledge about the different effect of the Tnc domains might be used to create artificial three-dimensional environments for cell transplantation. Hydrogels spiked with Tnc-domains might represent a promising tool in regenerative medicine.

## 1 Introduction

**FIGURE 1:**
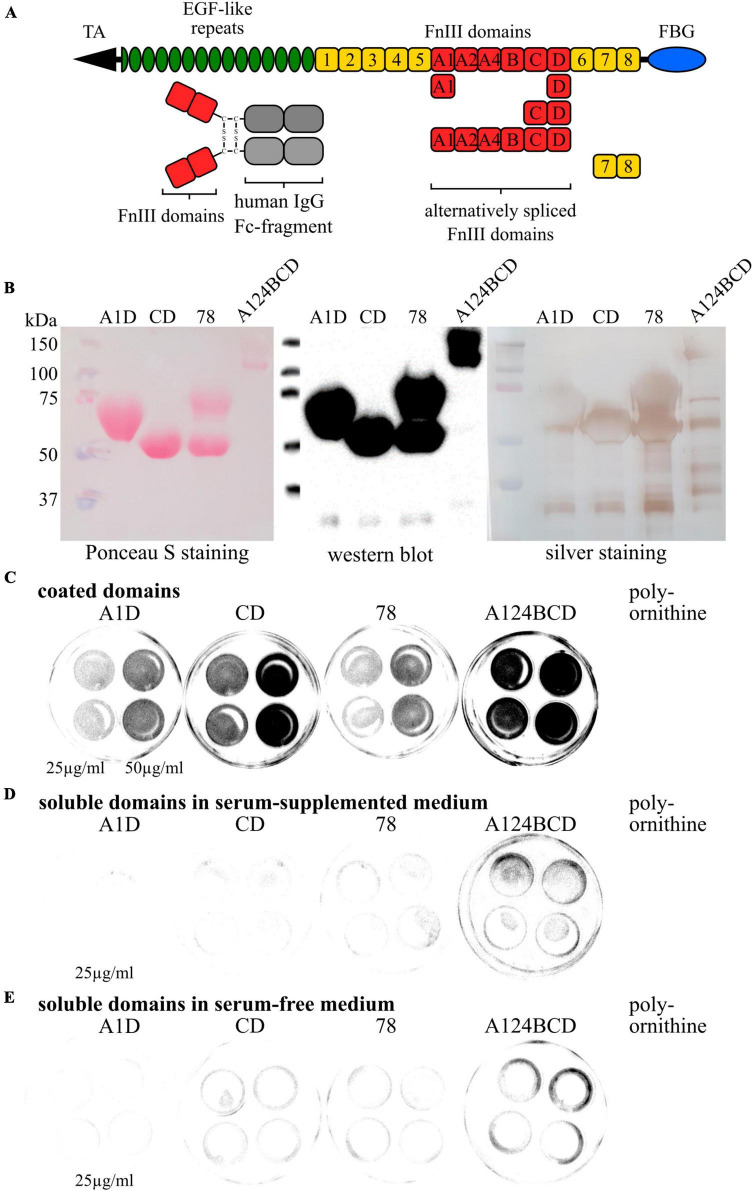
Characterization of purified Fc recombinants and coating test. **(A)** Schematic overview of a Tnc monomer with the location of the analyzed FnIII domains and their link to the human IgG Fc fragment. **(B)** Examination of the purity of the produced Fc recombinants via Ponceau S staining, western blot analysis and silver staining. **(C)** Coating test with coated domains on cell culture dishes. Fc-constructs were visualized by the anti-Fc-antibody. **(D)** Coating test with soluble domains in serum-supplemented medium on cell culture dishes. Fc-constructs were visualized by the anti-Fc-antibody. **(E)** Coating test with soluble domains in serum-free medium on cell culture dishes. Fc-constructs were visualized by the anti-Fc-antibody.

In the developing brain neural stem cells, the so-called radial glial cells, proliferate to expand the cell mass and give rise to the three neural cell types of the central nervous system (CNS), namely neurons, astrocytes and oligodendrocytes (Dimou and Götz, 2014; Taverna et al., 2014). This follows a strictly ordered process and is directed by extrinsic and intrinsic factors (Martynoga et al., 2012). Numerous guidance cues have been described. Among growth factors and cell-cell-interaction, the cell-matrix-interaction has been shown to play a crucial role in the determination of the cell fate (Theocharidis et al., 2014; Reinhard et al., 2016; Faissner et al., 2017). The extracellular matrix (ECM) is a three-dimensional (3D) scaffold consisting of glycoproteins and proteoglycans. It structures the pericellular environment and is not only important for the physical stability of the tissue, but also for signal transduction (Hynes and Naba, 2012; Naba et al., 2012, 2016). One promising protein in this context is the glycoprotein tenascin-C (Tnc). It is expressed during development and in the adult neural stem cell niches (Faissner et al., 2017). Furthermore, it is upregulated in tumors and after injury (Roll et al., 2012; Brösicke and Faissner, 2015; Roll and Faissner, 2019). In mouse, its structure is a hexabrachion whereby one monomer consists of a cysteine-rich amino terminal assembly domain (TA), 14.5 epidermal growth factor (EGF)-like repeats, eight constitutively expressed fibronectin type III (FnIII) domains (1–8), six alternatively spliced FnIII domains, namely A1, A2, A4, B, C, D, between the 5th and 6th FnIII domain and a fibrinogen (FBG) terminus (Jones and Jones, 2000; Giblin and Midwood, 2015). Through the binding of several receptors, like different integrins, the EGF-receptor or the receptor-type protein tyrosine phosphatase beta/zeta (RPTPβ/ζ), Tnc is able to activate different intracellular signaling pathways, like the focal adhesion kinase (FAK), the mitogen-activated protein kinase (MAPK) signaling pathway and the phosphatidylinositol 3-kinase (PI3K)/Akt pathway (Garwood et al., 2001; Swindle et al., 2001; Czopka et al., 2010; Tucker and Chiquet-Ehrismann, 2015; Jarocki et al., 2019). Thereby, Tnc can influence the behavior and the fate of the cells, like neurite outgrowth, oligodendrocyte precursor cell (OPC) maturation and cell migration (Husmann et al., 1992; Czopka et al., 2009, 2010; Jarocki et al., 2019; Schaberg et al., 2022). Due to the alternatively spliced FnIII domains up to 64 isoforms can be generated theoretically in mice, of which 27 isoforms are found to be expressed in the developing brain and 20 isoforms are produced by cortical neurospheres *in vitro* (Joester and Faissner, 1999, 2001; von Holst et al., 2007; Theocharidis et al., 2012). Among others, the domain combinations A1D, CD, and A124BCD are detected. The neurosphere model is an established method for the

cultivation of neural stem and progenitor cells (NSPCs) (Reynolds and Weiss, 1992; Louis et al., 2013). Using this model, we wanted to identify the impact of the Tnc-derived FnIII domains A1D, CD, A124BCD, and 78 on the fate and behavior of NSPCs. The FnIII domain 78 is located within the constitutively expressed region of Tnc and neither supports adhesion, nor repulsion of glia cells. Therefore, it was chosen as appropriate neutral control (Scholze et al., 1996). Since Tnc can be cleaved within the alternatively spliced segments by the matrix metalloproteinases (MMP) 2, 3, and 7, corresponding active single FnIII domain fragments might be created *in vivo* (Siri et al., 1995; Bell et al., 1999). Protein fragments with biological activities are called matricryptines and their activity can differ from the effect of the full-length protein (Ricard-Blum and Salza, 2014). We produced the domains in Chinese hamster ovary (CHO) cells. The vector construct caused the expression of the domains in dimerized form by fusing them to the Fc-fragment of a human immunoglobulin G (IgG) antibody, which ensured an easy purification and a reliable detection (see Figure 1A; Rigato et al., 2002). The production in eukaryotic cells had the advantage that the domains were glycosylated. We cultured the NSPCs derived from murine neurospheres in presence of the domains, either provided as coated culture substrates or as soluble additives *in vitro* and analyzed differentiation, proliferation and migration of the cells.

## 2 Materials and methods

### 2.1 Production of the Fc constructs

Tnc constructs were produced in transfected CHO cells created by Rigato et al. (2002). The expression and purification of the Fc recombinants was carried out as described by Rigato et al. (2002) with minor modifications. The cells were stored in liquid nitrogen. An aliquot was thawed in a water bath at 37°C for 1 min and transferred into fresh, warm CHO medium containing F12 (Gibco, Thermo Fisher Scientific, Waltham, MA, USA, Cat# 21765-029), 10% (v/v) fetal calf serum (FCS; Gibco, Thermo Fisher Scientific, Waltham, MA, USA, Cat# 10270-106) and 0.1% (v/v) Gentamicin (Sigma-Aldrich, St. Louis, MO, USA, Cat# G1397). After centrifugation the cells were resuspended in CHO medium and cultured in 10 cm dishes (Sarstedt, Nümbrecht, Germany, Cat# 83.3902) at 37°C and 6% CO_2_ under selection pressure from G418 (Sigma-Aldrich, St. Louis, MO, USA, Cat# G8168; starting with 1 mg/ml and maintaining at 0.1 mg/ml). The medium was changed every 3–4 days. When the cells reached 80% confluency, they were split by detachment with 0.05% (w/v) trypsin-EDTA (Ethylenediaminetetraacetic acid) (T/E; Gibco, Thermo Fisher Scientific, Waltham, MA, USA, Cat# 25300-062) for 5 min. The digestion was stopped via the addition of CHO medium. After centrifugation a portion of cells was transferred into a new dish with fresh CHO medium. For the production of the Fc constructs, the cells were split into T225 flasks (Thermo Fisher Scientific, Waltham, MA, USA, Cat# 159934) or roller bottles (Corning, Corning, NY, USA, Cat# 430849) with 50 ml or 100 ml selection medium, respectively (CHO medium containing 0.1 mg/ml G418), and grew until they reached a confluency of 60%. Then, the medium was replaced by 25 or 75 ml, respectively, production medium containing F12, 1% (v/v) Ultra low IgG FCS (Gibco, Thermo Fisher Scientific, Waltham, MA, USA, Cat# A33819-01), 0.1% (v/v) Gentamicin and 10 mM sodium butyrate (Merck, Darmstadt, Germany, Cat# 8.17500.0100). After 2 days the conditioned medium was collected and replaced by fresh production medium. The collected medium was centrifuged and the supernatant was mixed with 0.05% (w/v) sodium azide. After 1–2 additional days the second production medium was collected and treated like the first. For the purification protein A-sepharose columns were prepared by washing 1.5 g protein A-sepharose (Cytiva, Freiburg im Breisgau, Germany, Cat# 17078001) with water for 5 h at 4°C on a rocker, changing the water every hour. In an additional washing step, the sepharose was incubated in phosphate-buffered saline (PBS; 137 mM sodium chloride, 3 mM potassium chloride, 6.5 mM disodium hydrogen phosphate, 1.5 mM potassium dihydrogen phosphate; pH 7.4) over night at 4°C on a rocker. The next day, the PBS-protein A-sepharose mixture was divided equally on five columns (Bio-Rad, Feldkirchen, Germany, Cat# 732-1010) and remained at 4°C for the whole purification process. Prior to the purification, the columns were washed twice with 25 ml PBS. About 200–250 ml conditioned production medium was purified in one step. The flow-through was collected for further purifications. The columns were washed twice with 25 ml PBS and incubated with 6 ml 0.1 M glycine (pH 2.7) for 45 min. The conditioned glycine eluate was collected and the columns were washed twice with PBS and stored with PBS containing 0.025% (w/v) sodium azide at 4°C. The purified eluate of several purifications was dialyzed with the Servapor dialysis tubing (Serva, Heidelberg, Germany, Cat# 44145.04) in the 100-fold volume of PBS at 4°C for 24 h with the PBS being changed thrice. Afterward, the dialyzed eluate was concentrated with a Pierce^®^ Concentrator (Thermo Fisher Scientific, Waltham, MA, USA, Cat# 87751). The concentration of the purified protein constructs was measured with the Pierce BCA Protein Assay Kit (Thermo Fisher Scientific, Waltham, MA, USA, Cat# 23225).

### 2.2 Detection and verification of the Fc recombinants

The Fc construct content in the conditioned medium, the purified flow-through and the eluate was detected using a dot blot. Therefore, 10 μl of the sample was dropped onto a nitrocellulose membrane (Bio-Rad, Feldkirchen, Germany, Cat# 162-0146) and blocked with 5% (w/v) low fat milk powder in 1xTBST [Tris-buffered saline (25 mM Tris–HCl, pH 8.0, 150 mM NaCl) supplemented with 0.05% (v/v) Tween 20] for 1 h. The anti-human IgG Fc antibody was diluted 1:7000 [goat; horseradish peroxidase (HRP)-coupled; Sigma-Aldrich; Cat# A0170; RRID:AB_257868] in the blocking solution and the membrane was incubated with the antibody for 1 h. After two washing steps with 1xTBST and one washing step with 1xTBS (Tris-buffered saline) the membrane was developed with the Clarity™ Western ECL Substrate from Bio-Rad (Feldkirchen, Germany, Cat# 1705061) and visualized with the MicroChemi chemiluminescence device with the Gel Capture Software by biostep (Burkhardtsdorf, Germany; Version: 6.6).

To proof the successful purification of the Fc constructs, sodium dodecyl sulfate polyacrylamide gel electrophoresis (SDS-PAGE) with western blot analysis and Ponceau S staining as well as silver staining were performed. For the SDS-PAGE 6 μl sample were mixed with 2 μl fourfold sample buffer [250 mM Tris–HCl (pH = 6.8), 9.2% (w/v) SDS, 40% (v/v) glycerol, 20% (v/v) mercaptoethanol, 0.3 mM bromophenol blue] and denaturized for 5 min at 95°C. The samples were loaded onto a 10% (v/v) polyacrylamide gel containing SDS and the proteins were separated with 15 mA. Afterward, the proteins were transferred to a polyvinylidene difluoride (PVDF) membrane (Roth, Karlsruhe, Germany, Cat# T830.1) by western blotting for 1 h at 75 mA. The protein content on the membrane was visualized with Ponceau S staining [0.25% (w/v) Ponceau S (Serva, Heidelberg, Germany, Cat# 33429), 40% (v/v) methanol, 15% (v/v) glacial acetic acid]. After documentation the dye was washed out with water and the membrane was blocked with 5% (w/v) milk powder in 1xTBST for 1 h. Subsequently, the membrane was incubated with the anti-human IgG Fc antibody (1:7000; HRP-coupled) in the blocking solution for 1 h. The membrane was washed thrice with 1xTBST and once with 1xTBS. The Fc-fragment was detected and visualized as described above (see dot blot detection).

Additionally, coated and soluble Fc constructs were detected with the anti-human IgG Fc antibody and the Clarity™ Western ECL Substrate by Bio-Rad following the same instructions as before (see dot blot detection). The coated Fc recombinants were visualized on cell culture dishes (Greiner Bio-One, Kremsmünster, Austria, Cat# 627170) with the MicroChemi chemiluminescence device with the Gel Capture Software by biostep.

For the silver staining the gel was fixed twice after SDS-PAGE in fixing solution [30% (v/v) ethanol and 10% (v/v) glacial acetic acid] for 15 min each. Afterward, the incubation in reducing solution [15% (v/v) ethanol, 1.25% (v/v) sodium acetate solution, 0.5% (v/v) sodium thiosulfate solution] followed for 1 h. The gel was washed thrice in water for 10 min each and incubated with the silver solution [0.1% (w/v) AgNO_3_, 0.025% (v/v) formaldehyde] for 30 min. After 2 short washing steps with water, the gel was developed with a developer solution [2.5% (w/v) Na_2_CO_3_, 0.025% (v/v) formaldehyde] until a coloring became visible. The reaction was stopped with 1% (v/v) glacial acetic acid.

### 2.3 Isolation of embryonic neural stem and progenitor cells and neurosphere cultivation

The cells used in the experiments were isolated from NMRI mice (Charles River Laboratories; Cat# 605NMRI; RRID:IMSR_CRL:605). The mice had access to food and water *ad libitum* and were housed with a constant 12 h light-dark cycle. The European Council Directive of 22 September 2010 (2010/63/EU) for care of laboratory animals was obeyed. The mice handling and housing was supervised by the animal welfare commissioner of Ruhr University. Embryos of both sexes were used. Pregnant mice at embryonic day (E) 13 were sacrificed by cervical dislocation. The abdomen was opened and the uteri were transferred into 10 cm dishes with sterile PBS. The embryos were isolated and the age was determined based on the Theiler criteria (Theiler, 2013). The mice were decapitated and the heads were transferred into new dishes filled with sterile Minimum Essential Medium Eagle (MEM; Sigma-Aldrich, St. Louis, MO, USA, Cat# M7278). The brain was freed, the hindbrain was removed and the hemispheres were divided. The diencephalon and the mesencephalon were removed together with the olfactory bulbs. Afterward, the meninges were peeled off and the hippocampus and the ganglionic eminences were detached. The isolated cortices were collected in 2 ml reaction tubes filled with 1 ml sterile MEM. For the enzymatic digestion of the tissue 30 U/ml papain (Worthington, Lakewood, NJ, USA, Cat# LS003126) was solved in 1 ml MEM and supplemented with 40 μg/ml DNaseI (Worthington, Lakewood, NJ, USA, Cat# LS002007) and 240 μg/ml L-cysteine (Sigma-Aldrich, St. Louis, MO, USA, Cat# A9165). Before use, the mixture was sterile filtered (Millex-GV filter, 0.22 μm; Merck Millipore, Billerica, MA, USA, Cat# SLGV013SL). The tissue was incubated with the digestion solution at 37°C for 25 min. The digestion was stopped with ovomucoid [L-15 medium (Sigma-Aldrich, St. Louis, MO, USA, Cat# L5520), 1 mg/ml trypsin inhibitor (Sigma-Aldrich, St. Louis, MO, USA, Cat# T6522), 50 μg/ml bovine serum albumin (BSA; Sigma-Aldrich, St. Louis, MO, USA, Cat# A9418), 40 μg/ml DNase I] and the cells were separated by trituration. After centrifugation at 80 rcf for 5 min, the cell pellet was resuspended in fresh neurosphere medium containing Dulbecco’s Modified Eagle’s Medium (DMEM; Sigma-Aldrich, St. Louis, MO, USA, Cat# D6546) and F12 (Sigma-Aldrich, St. Louis, MO, USA, Cat# N4888) in a ratio of 1:1 supplemented with 0.2 mg/ml L-glutamine (Sigma-Aldrich, St. Louis, MO, USA, Cat# G7513), 1x B27 (Gibco, Thermo Fisher Scientific, Waltham, MA, USA, Cat# 17504-044) and 100 μg/ml penicillin/streptomycin (Sigma-Aldrich, St. Louis, MO, USA, Cat# P4333). The cells were cultured in a density of 400000 cell in 4 ml neurosphere medium supplemented with 20 ng/ml fibroblast growth factor 2 (FGF2, Peprotech, Cranbury, NJ, USA, Cat# 100-18B), 20 ng/ml EGF (Peprotech, Cranbury, NJ, USA, Cat# AF100-15) and 0.5 U/ml heparin (Sigma-Aldrich, St. Louis, MO, USA, Cat# H3149) in T25 flasks (Sarstedt, Nümbrecht, Germany, Cat# 83.3910.002) for 5 days at 37°C and 6% CO_2_.

### 2.4 Differentiation and proliferation assay under differentiation conditions

After 5 days prepared NSPCs formed neurospheres. These neurospheres were transferred into 15 ml centrifuge tubes and pelleted at 80 rcf for 5 min. Spheres were resuspended in T/E and digested at 37°C for 5 min. The digestion was stopped by addition of the same volume of ovomucoid and cells were separated by gentle trituration. After centrifugation, cells were resuspended in 1 ml neurosphere medium. For immunocytochemical (ICC) staining cells were plated in a density of 25000 cells per well on poly-L-ornithine (15 μg/ml; Sigma-Aldrich, St. Louis, MO, USA, Cat# P3655) coated 4 well dishes in differentiation medium (neurosphere medium supplemented with 1% FCS). For ribonucleic acid (RNA) isolation and protein samples cells were plated in a density of 500000 cells per dish (diameter 35 mm; Greiner Bio-One, Kremsmünster, Austria, Cat# 627160). The produced FnIII Fc constructs of Tnc were presented to the cells in two different ways: On the one hand, cell culture dishes were additionally coated with 25 μg/ml domains and on the other hand, 25 μg/ml domains were added soluble in the medium. For the control, an additional coating was omitted, or PBS was added to the medium, respectively. NSPCs were cultured at 37°C and 6% CO_2_ for 3 or 7 days *in vitro* (div).

### 2.5 Time-lapse video microscopy under proliferating conditions

For time-lapse video microscopy, the neurospheres were dissociated as described in section “2.4 Differentiation and proliferation assay under differentiation conditions.” The NSPCs were plated in a density of 30000 cells per well on poly-L-ornithine (15 μg/ml) coated 24 well plates (Corning, Corning, NY, USA, Cat# 353226) in neurosphere medium. After 2 h, the cells adhered and the medium was supplemented with EGF, FGF2 and heparin in the same concentrations as in the neurosphere culture to ensure proliferation conditions. For the analysis, the produced FnIII Fc recombinants were presented to the cells as coated substrates or as soluble medium additives, both with a concentration of 25 μg/ml. A total of 2 h after seeding the plate was placed in the video microscope system of an Axiovert 200 M provided with an AxioCam HRm and AxioVision-4.8.1 software (Carl Zeiss, Oberkochen, Germany). Additionally, two regulating elements, namely the “Tempcontrol 37-2 digital” and the “CTI-Controller 3700 digital” (PeCon GmbH, Erbach, Germany) ensured stable temperature and pH conditions. Within the system the cells were cultured at 37°C and 5% CO_2_. For analysis, five spots per domain combination were chosen and a picture was taken every 6 min for 96 h.

### 2.6 Immunocytochemistry of the differentiation and proliferation assay under differentiation conditions

The differentiation of the NSPCs was evaluated via an ICC staining of different cell type specific markers. The wells were washed with KRH/A [Krebs-Ringer solution, HEPES (4-(2-hydroxyethyl)-1-piperazineethanesulfonic acid)-buffered (125 mM NaCl, 4.8 mM KCl, 1.3 mM CaCl_2_*2H_2_O, 1.2 mM MgSO_4_*7H_2_O, 1.2 mM KH_2_PO_4_, 5.6 mM D-glucose, 25 mM HEPES) supplemented with 0.1% (w/v) BSA (Sigma-Aldrich, St. Louis, MO, Cat# A7930)]. The antibody O4 [1:50; mouse; IgM; hybridoma clone 81 (Sommer and Schachner, 1981)] which binds to a glycolipid on the cell surface of oligodendrocytes was diluted in KRH/A and incubated on living cells. After 15 min these wells were washed twice with KRH (Krebs-Ringer solution, HEPES-buffered). Subsequently, all cells were fixed with 4% (v/v) paraformaldehyde (PFA) for 10 min. The cells were permeabilize with PBS supplemented with 0.1% (v/v) Triton X-100 and 1% (w/v) BSA (PBST). The following antibodies were diluted in PBST and incubated for 1 h at room temperature: anti-glial fibrillary acidic protein (GFAP; 1:300; rabbit; Agilent; Cat# Z0334; RRID:AB_10013382), anti-βIII-tubulin (1:300; mouse, IgG; Sigma-Aldrich; Cat# T8660; RRID:AB_477590), anti-phospho histone H3 (PH3; 1:100; rabbit; IgG; Millipore; Cat# 06-570; RRID:AB_310177) and anti-nestin (1:500; mouse; IgG; Millipore; Cat# MAB353; RRID:AB_94911; see Supplementary Table 1). The wells were washed thrice with PBS supplemented with 0.1% (w/v) BSA (PBS/A) and the following secondary antibodies were diluted in PBS/A: anti-rabbit (1:300; goat; IgG; Cy2-coupled; Jackson ImmunoResearch Labs; Cat# 111-545-045; RRID:AB_2338049) and anti-mouse (1:300; goat; IgG; Cy3-coupled; Jackson ImmunoResearch Labs; Cat# 115-165-068; RRID:AB_2338686; see Supplementary Table 2) as well as the nuclei marker Hoechst (bisBenzimide H 33258, 1:100000; Sigma-Aldrich, St. Louis, MO, USA, Cat# B2883). The secondary antibodies were incubated under exclusion of light for 1 h. Afterward, the wells were washed twice with PBS and mounted with 50% (v/v) PBS and 50% (v/v) glycerol. The experiments were performed in biological triplicates and for one N six pictures were taken with the Axioplan 2 imaging from Zeiss (Oberkochen, Germany) at 200-fold magnification (*N* = 3, *n* = 18). The marker-positive cells were counted with the “cell counter” plug-in of the software ImageJ and the percentage of these cells was determined with respect to the total number of Hoechst-positive nuclei.

### 2.7 Tracking of cell divisions in the time-lapse videos

The videos were observed frame by frame and every cell division was documented. The collectivity of cell division events in the videos were counted and divided by the number of quantified cells. It must be noted that only visible cell divisions could be counted and in case of sphere formation the divisions were not observable in total anymore. Three biological independent replicates with five videos each were accomplished and the population of cells seen on the first frame of the video was tracked (*N* = 3). Thereby, different numbers of cells were quantified for each condition, namely the following: coated FnIII A1D (cA1D) *n* = 204, coated FnIII CD (cCD) *n* = 186, coated FnIII 78 (c78) *n* = 222, coated FnIII A1A2A4BCD (cA124BCD) *n* = 233, poly-L-ornithine control *n* = 192, soluble FnIII A1D (sA1D) *n* = 164, soluble FnIII CD (sCD) *n* = 169, soluble FnIII 78 (s78) *n* = 174, soluble FnIII A1A2A4BCD (sA124BCD) *n* = 129, PBS control *n* = 208.

### 2.8 Migration analysis in the time-lapse videos

The distance covered by each cell in the video was measured by using the “Manual Tracking” plug-in of the software ImageJ. In this process, the pixel size was determined as 0.645 μm per pixel and the time period between each frame as 6 min. This allowed the calculation of the covered distance of a cell per day to include also the cells which died or emigrated out of the video during the evaluated time period. The experiment was performed thrice with five videos each (*N* = 3). The cells seen in the first frame of each video were tracked, resulting in following cell numbers: cA1D *n* = 242, cCD *n* = 181, c78 *n* = 223, cA124BCD *n* = 223, poly-L-ornithine control *n* = 193, sA1D *n* = 164, sCD *n* = 171, s78 *n* = 176, sA124BCD *n* = 129, PBS control *n* = 209.

### 2.9 RNA isolation, cDNA synthesis, and PCR analysis

The messenger (m) RNA isolation was performed with the GenElute Mammalian Total RNA Miniprep Kit (Sigma-Aldrich, St. Louis, MO, Cat# RTN350-1KT) following the instructions in the manual. The complementary deoxyribonucleic acid (cDNA) was synthesized with the First-Strand cDNA Synthesis Kit (Fermentas, Waltham, MA, USA, Cat# K1612) following the instructions in the manual. The cDNA was stored at −20°C. The gene expression was semi-quantitatively analyzed with polymerase chain reaction (PCR) using the primers listed in Supplementary Table 3. The housekeeping gene *Actb* was used for normalization. The master mix for one sample contains 2.5 μl 10-fold PCR buffer (Sigma-Aldrich, St. Louis, MO, USA, Cat# P2192), 0.5 μl dNTP (Roth, Karlsruhe, Germany, dTTP: Cat# K036.1, dCTP: Cat# K038.1, dGTP: Cat# K037.1, dATP: Cat# K035.1), 0.5 μl (5 pMol) of each primer (see Supplementary Table 3), 0.25 μl *Taq*-polymerase (Sigma-Aldrich, St. Louis, MO, USA, Cat# D6677), 19.75 μl H_2_O and 1 μl cDNA. The PCR was performed with the Mastercycler nexus X2 from Eppendorf (Hamburg, Germany) using the following program: initial denaturation (90°C for 2 min 40 s), denaturation (90°C for 30 s), annealing (primer specific annealing temperature see Supplementary Table 3, for 30 s), elongation (72°C for 40 s) and final elongation (72°C for 5 min). The cycle number was primer specific (see Supplementary Table 3). Amplicons were supplemented with sixfold sample buffer [40 mM TAE (Tris-acetate-EDTA), 10 mM EDTA, 15% (v/v) glycerol, 0.25% (w/v) bromophenol blue] and separated within a 1.5% agarose (Sigma-Aldrich, St. Louis, MO, USA, Cat# A9539) gel at 120 V for 30 min. The results were detected with the imaging platform Essential V6 from Uvitec Cambridge (Cambridge, UK). The band intensity was evaluated with ImageJ.

### 2.10 Protein analysis via SDS-PAGE and western blot analysis

Protein samples were collected using radioimmuno- precipitation assay buffer [RIPA; 10 mM Tris–HCl (pH 8.0), 1 mM EDTA, 0.5 mM ethylene glycol-bis(β-aminoethyl ether)-N,N,N′,N′-tetraacetic acid (EGTA), 1% (v/v) Triton X-100, 0.1% (w/v) sodium deoxycholate, 0.1% (v/v) SDS and 140 mM NaCl] supplemented with 1% (v/v) protease inhibitor phenylmethylsulfonyl fluoride (PMSF; solved 17.4 mg/ml in methanol; MP Biomedicals, Irvine, CA, USA, Cat# 195381) and 1% (v/v) aprotinin (APR; Sigma-Aldrich, St. Louis, MO, USA, Cat# 10236624001). Cells were detached from the dish using a cell scraper (Sarstedt, Nümbrecht, Germany, Cat# 83.1832). For SDS-PAGE, 12 μl protein sample was mixed with 3 μl fourfold sample buffer and heated at 95°C for 5 min. SDS-PAGE and western blot was performed as described in section “2.2 Detection and verification of the Fc recombinants.” For protein detection the membranes were incubated at 4°C over night on a rocker with following primary antibodies: anti-phospho extracellular signal-regulated kinases (pErk; 1:1000; rabbit; Cell Signaling Technology; Cat# 9101; RRID:AB_331646), anti-total Erk (tErk; 1:1000; mouse; Santa Cruz Biotechnology; Cat# sc-271269; RRID:AB_10611091), anti-phospho Akt (pAkt; 1:2000; rabbit; IgG; Cell Signaling Technology; Cat# 4060; RRID:AB_2315049), anti-total Akt (tAkt; 1:1000; rabbit; IgG; Cell Signaling Technology; Cat# 4691; RRID:AB_915783), anti-phospho focal adhesion kinase (pFAK; 1:1000; rabbit; Cell Signaling Technology; Cat# 3283; RRID:AB_2173659), anti-total FAK (tFAK; 1:1000; rabbit; Cell Signaling Technology; Cat# 3285; RRID:AB_2269034), anti-notch (1:1000; rabbit; IgG; Cell Signaling Technology; Cat# 3608; RRID:AB_2153354), anti-cleaved notch (1:1000; rabbit; IgG; Cell Signaling Technology; Cat# 4147; RRID:AB_2153348), anti-Sam68 (Src associated in mitosis, of 68 kDa; 1:500; mouse; IgG; Santa Cruz Biotechnology; Cat# sc-1238; RRID:AB_627858) and anti-α-tubulin (1:10000; mouse; IgG; Sigma-Aldrich; Cat# T9026; RRID:AB_477593; see Supplementary Table 1) diluted in 5% (w/v) milk powder in 1xTBST. The membranes were washed thrice with 1x TBST and incubated at room temperature for 1 h on a rocker with following HRP-coupled secondary antibodies: anti-mouse [1:5000 (1:10000 for α-tubulin); goat; IgG; Jackson ImmunoResearch Labs; Cat# 115-035-068; RRID:AB_2338505] and anti-rabbit (1:5000; goat; IgG; Jackson ImmunoResearch Labs; Cat# 111-035-144; RRID:AB_2307391; see Supplementary Table 2) diluted in 5% (w/v) milk powder in 1xTBST. Before detection (see section “2.2 Detection and verification of the Fc recombinants”) the membranes were washed thrice with 1xTBST and twice with 1xTBS. For quantification the intensity of the bands was measured with ImageJ. The total proteins served as reference for phosphorylated or cleaved proteins, whereas Sam68 was normalized to α-tubulin.

### 2.11 Statistical analysis

The statistical analysis of the results was done with the software GraphPad Prism (Version 5.02; GraphPad Software; San Diego, CA, USA). The results were presented as mean ± SEM (standard error of the mean). The results were normalized to the control by dividing every value through the mean of the control. The control was set as a baseline of 100% so that the results were shown in relation to the control. The Shapiro-Wilks test was used to determine the normality distribution of the data sets. The differences within one group was quantified with an ANOVA (analysis of variants) with a *post-hoc* Bonferroni’s test or a Kruskal-Wallis with the *post-hoc* Dunn’s test, if the data sets were not distributed normally. The following *p*-values were defined as significant: *p ≤ 0.05, **p ≤ 0.01, and ***p ≤ 0.001. All experiments were performed in biological triplicates (*N* = 3). All values were summarized in Supplementary Tables 4–7.

## 3 Results

### 3.1 Characterization of purified Fc constructs

The produced FnIII domains of Tnc, namely A1D, CD, 78 and A124BCD which were linked to the Fc fragment were characterized by SDS-PAGE, followed by silver staining and western blotting with Ponceau S staining and detection of the Fc constructs with a specific antibody (see Figure 1B). The silver staining and the Ponceau S staining detected all proteins in the samples, whereas the antibody only discovered the Fc constructs. The results revealed pure samples of the FnIII A1D and CD domain including proteins with a molecular weight of 50–75 kDa. The sample of the 78 domain displayed two protein fractions on the gel and membrane with a weight of 50 and 75 kDa which were both detectable with the anti-Fc antibody, indicating that both fractions were Fc constructs. These fragments might be two different tertiary structures of the recombinant. Additionally, varying weights of one construct might be attributed to different glycosylation forms of the domains. It is assumed that the A1D domain combination contained three N-glycosylation sites and five O-glycosylation sites resulting in an increased mass compared to the CD domain combination, which only includes one N-glycosylation site and three O-glycosylation sites (Giblin and Midwood, 2015). The constitutively expressed domain 78 contained one N-glycosylation site and one O-glycosylation site and might be produced by the CHO cells as an unglycosylated and as a twice glycosylated protein. The sample containing the A124BCD domain combination showed in the Ponceau S staining two bands at heights of 100 kDa and 150 kDa. The more sensitive silver staining revealed additional bands with a weight between 50 and 75 kDa and between 37 and 50 kDa. The A124BCD domain combination was the longest fragment including six alternatively spliced FnIII domains. The additional bands in the silver staining might be single domains or smaller domain combinations that were produced or spliced by the CHO cells. However, the antibody detected only the proteins with a molecular weight of 100 and 150 kDa indicating that this were the produced Fc constructs. Again, different degrees of glycosylation of the fragment with eleven N-glycosylation sites and six O-glycosylation sites might explain the two bands (Midwood et al., 2016). Additionally, all samples displayed bands at a height below 37 kDa which were also detected with the antibody. These proteins might be the Fc fragments without FnIII domains. Additionally, the coated Fc constructs on cell culture dishes were visualized with the anti-Fc antibody. Therefore, the recombinants were coated with a concentration of 25 and 50 μg/ml. The coated constructs from each domain were detectable, showing a difference in intensity at the two different concentrations (see Figure 1C). Furthermore, after the addition of soluble domains into serum-supplemented and serum-free medium the signal was distinctly decreased. This indicates that the domains stayed soluble and did not adhere to the plate during cultivation (see Figures 1D, E).

### 3.2 The soluble Tnc-derived domain combination A124BCD regulated NSPCs differentiation, while the coated domain A1D influenced astrocyte differentiation

**FIGURE 2:**
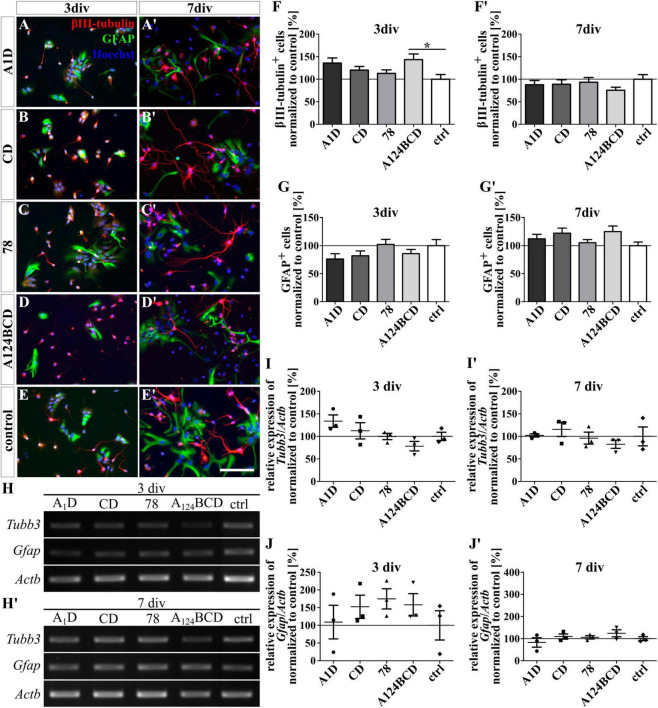
Differentiation assay of neurons and astrocytes treated with the soluble FnIII domains of Tnc. **(A–E’)** Exemplary images of the ICC for βIII-tubulin-positive neurons (red) and GFAP-positive astrocytes (green). Nuclei were stained with Hoechst (blue). **(F–G’)** Quantitative evaluation of the percentage of βIII-tubulin-positive and GFAP-positive cells normalized to the control. The mean of the control was set as a baseline of 100%. **(H,H’)** Exemplary images of the PCR for *Tubb3*, *Gfap*, and *Actb*. **(I–J’)** Evaluation of the relative expression of the gene *Tubb3* and *Gfap* in relation to the expression of *Actb*. The values were normalized to the control which was set as a baseline of 100%. [scale bar: 100 μm; div = days *in vitro*; mean ± SEM; ICC: *N* = 3, *n* = 18; **(F,F’,G)** ANOVA with *post-hoc* Bonferroni’s test, **(G’)** Kruskal–Wallis test with *post-hoc* Dunn’s test; PCR: *N* = 3, *n* = 3; **(I–J’)** Kruskal–Wallis test with *post-hoc* Dunn’s test; *p ≤ 0.05].

**FIGURE 3:**
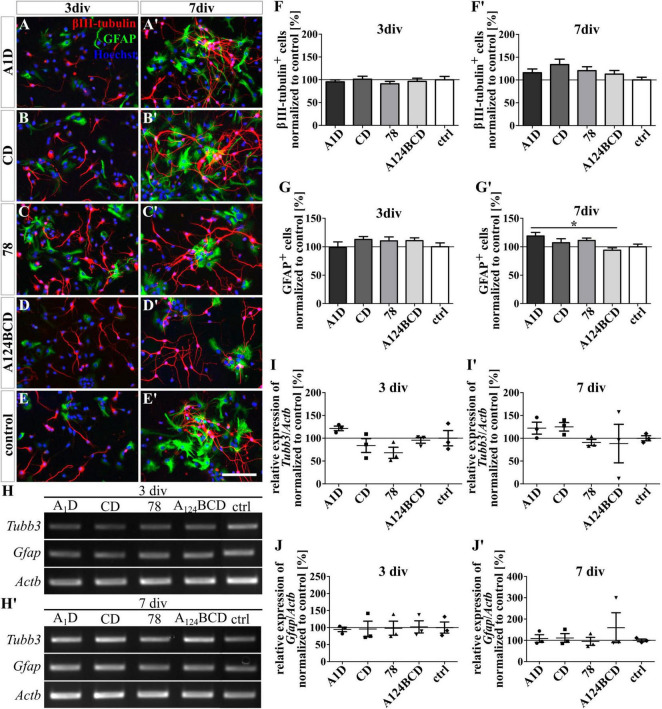
Differentiation assay of neurons and astrocytes cultured on coated FnIII domains of Tnc. **(A–E’)** Exemplary images of the ICC for βIII-tubulin-positive neurons (red) and GFAP-positive astrocytes (green). Nuclei were stained with Hoechst (blue). **(F–G’)** Quantitative evaluation of the percentage of βIII-tubulin-positive and GFAP-positive cells normalized to the control. The mean of the control was set as a baseline of 100%. **(H,H’)** Exemplary images of the PCR for *Tubb3*, *Gfap*, and *Actb*. **(I-J’)** Evaluation of the relative expression of the gene *Tubb3* and *Gfap* in relation to the expression of *Actb*. The values were normalized to the control which was set as a baseline of 100%. [scale bar: 100 μm; div = days *in vitro*; mean ± SEM; ICC: *N* = 3, *n* = 18; **(F,G)** ANOVA with *post-hoc* Bonferroni’s test, **(F’,G’)** Kruskal–Wallis test with *post-hoc* Dunn’s test; PCR: *N* = 3, *n* = 3; **(I–J’)** Kruskal–Wallis test with *post-hoc* Dunn’s test; *p ≤ 0.05].

The influence of the alternatively spliced FnIII domains of Tnc A1D, CD, and A124BCD as well as of the constitutively expressed domain 78 on the differentiation of NSPCs was analyzed with an ICC staining. Additionally, the relative expression of marker genes was determined using PCR. Therefore, the domains were presented to the cells as coating or as soluble additive. The evaluation of the number of βIII-tubulin-positive neurons showed an increase of 43.4% of the neuron population in the cultures treated with the soluble domain A124BCD after 3 div compared to the control (sA124BCD 143.4 ± 12.5% vs. control 100 ± 10.4%, *p* ≤ 0.05; see Figures 2D–F). On the other hand, the cultivation of the NSPCs on the coated domain A124BCD for 3 days did not support the differentiation of neurons compared to the control [cA124BCD 96.6 ± 6.9% vs. control 100 ± 7.4%, not significant (n.s.); see Figures 3D–F]. However, the other soluble alternatively spliced FnIII domains, namely A1D and CD, as well as the constitutively expressed FnIII domain 78 did not cause an altered differentiation of neurons that differed from the control (see values in Supplementary Table 4 and see Figures 2A–F’). Furthermore, the neuron promoting effect of the soluble domain A124BCD was not detectable anymore after 7 div (sA124BCD 75.6% ± 6.7% vs. control 100% ± 10.2%, n.s.). This might be due to an early promotion of the differentiation of neurons in the first 3 days which reached a plateau after 7 div. Moreover, the coated domains seemed to have no effect on the differentiation of neurons after 3 and 7 div (see Figures 3A–F’; see values in Supplementary Table 4) underlining the importance of the mode of presentations. However, the PCR results revealed no significant differences in the expression of the marker gene *Tubb3* after the cultivation of NSPCs in the presence of coated (see Figures 3H–I’) or soluble FnIII domains (see Figures 2H–I’; see values in Supplementary Table 5) for 3 and 7 div. For PCR analysis a mixed culture was investigated. The neuron population made up one fifth of the culture, therefore a relative increase of 44% represented about 8% of the total cell population. This in conjunction with inter experimental variability probably is the reason why a potentially altered gene expression in neurons might blur within the mixed culture and not result in statistically significant differences.

The differentiation of GFAP-positive astrocytes was promoted by the coated domain A1D by 25% in comparison to the coated domain A124BCD (cA1D 119 ± 6.3% vs. cA124BCD 94.1 ± 4%, *p* ≤ 0.05; see Figures 3A’, D’, G’) after 7 div. In contrast, the soluble FnIII domain A1D (112.1% ± 8.1%) and any other soluble FnIII domain did not support the astroglial differentiation after 3 and 7 div (see Figures 2A–E’, G, G’). However, except for the coated FnIII domain A1D after 7 div, other coated FnIII domains seemed to have no influence on the astrocyte differentiation after 3 and 7 div (see Figures 3A–E’, G, G’; see values in Supplementary Table 4). Though, the evaluation of the relative expression of the marker gene *Gfap* via PCR indicated no significant differences within the cultures treated with the coated (see Figures 3H, H’, J, J’) or with the soluble FNIII domains (see Figures 2H, H’, J, J’ and see values in Supplementary Table 5) after 3 and 7 div. A heterogenous mixed culture was studied and the astrocytes represented a minor fraction. Therefore, as noted above, the altered gene expression might blur within the total cell population, rendering changes within the astrocyte population undetectable on a significance level.

**FIGURE 4:**
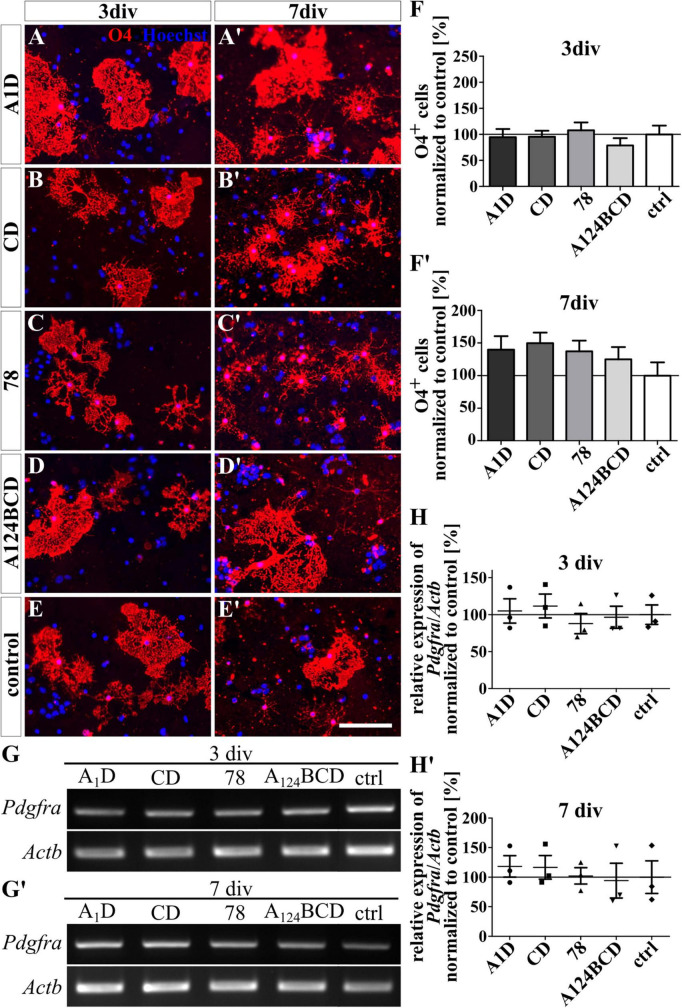
Differentiation assay of oligodendrocytes cultured on coated FnIII domains of Tnc. **(A–E’)** Exemplary images of the ICC for O4-positive oligodendrocytes (red). Nuclei were stained with Hoechst (blue). **(F,F’)** Quantitative evaluation of the percentage of O4-positive cells normalized to the control. The mean of the control was set as a baseline of 100%. **(G,G’)** Exemplary images of the PCR for *Pdgfra* and *Actb*. **(H,H’)** Evaluation of the relative expression of the gene *Pdgfra* in relation to the expression of *Actb*. The values were normalized to the control which was set as a baseline of 100%. [scale bar: 100 μm; div = days *in vitro*; mean ± SEM; ICC: *N* = 3, *n* = 18; **(F’)** ANOVA with *post-hoc* Bonferroni’s test, **(F)** Kruskal–Wallis test with *post-hoc* Dunn’s test; PCR: *N* = 3, *n* = 3; **(H,H’)** Kruskal–Wallis test with *post-hoc* Dunn’s test].

**FIGURE 5:**
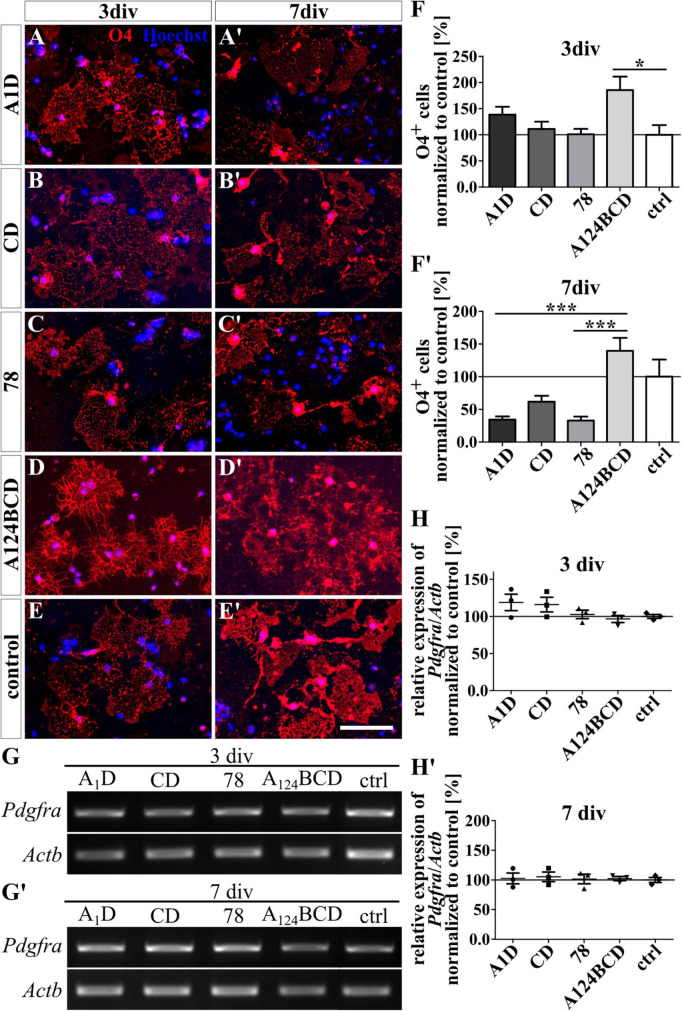
Differentiation assay of oligodendrocytes treated with soluble FnIII domains of Tnc. **(A–E’)** Exemplary images of the ICC for O4-positive oligodendrocytes (red). Nuclei were stained with Hoechst (blue). **(F,F’)** Quantitative evaluation of the percentage of O4-positive cells normalized to the control. The mean of the control was set as a baseline of 100%. **(G,G’)** Exemplary images of the PCR for *Pdgfra* and *Actb*. **(H,H’)** Evaluation of the relative expression of the gene *Pdgfra* in relation to the expression of *Actb*. The values were normalized to the control which was set as a baseline of 100%. [scale bar: 100 μ m; div = days *in vitro*; mean ± SEM; ICC: *N* = 3, *n* = 18; **(F,F’)** Kruskal–Wallis test with *post*-*hoc* Dunn’s test; PCR: *N* = 3, *n* = 3; (H,H’) Kruskal–Wallis test with *post*-*hoc* Dunn’s test; *p ≤ 0.05, ***p ≤ 0.001].

The coated FnIII domains seemed to have no influence on the differentiation of O4-positive oligodendrocytes after 3 and 7 div (see Figures 4A–F’ and see values in Supplementary Table 4). This was also confirmed by PCR which showed no significant differences in the relative expression of the oligodendrocyte marker gene *platelet-derived growth factor receptor alpha* (*Pdgfra*) (see Figures 4G–H’ and see values in Supplementary Table 5). In contrast, the soluble FnIII domain A124BCD increased significantly the number of oligodendrocytes by 86% compared to the control (sA124BCD 185.6 ± 25.9% vs. control 100 ± 18.4%, *p* ≤ 0.05) after 3 div (see Figures 5D–F). However, the other soluble domains did not cause this effect (see Figures 5A–C). Moreover, after 7 div the cultures treated with the soluble FnIII A124BCD domain gained a higher number of oligodendrocytes than the cultures treated with the soluble domains A1D and 78 (sA124BCD 139.6 ± 19.7% vs. sA1D 34.5 ± 4.8, *p* ≤ 0.001; sA124BCD 139.6 ± 19.7% vs. s78 32.8 ± 6.2%, *p* ≤ 0.001; see Figures 5A’, C’, D’, F’), but not compared to the FnIII domain CD or the control (see Figures 5B’, C’, E’, F’). While the soluble domain A124BCD promoted the oligodendrocyte differentiation compared to the control during an early time period, it might reach a plateau after some time, which explains why after 7 days there is no longer a significant difference between A124BCD and the control. When considering the unnormalized percentage of oligodendrocytes in the cultures treated with the soluble domains after 7 div, the number of oligodendrocytes in the cultures treated with the FnIII domain A124BCD (20 ± 2.8%) was more than four times the number of oligodendrocytes treated with the FnIII domains A1D (4.9 ± 0.7%) and 78 (4.7 ± 0.9%; see values in Supplementary Table 4). However, the PCR results revealed no significant differences in the relative expression of *Pdgfra* in the cultures treated with the soluble FNIII domains (see Figures 5G–H’ and see values in Supplementary Table 5). The oligodendrocyte population represented a twentieth to a fifth of the total population. Hence the difference population, similar to the situation with neurons and astrocytes discussed previously, amounted to a minor fraction and a potentially altered gene expression might blur within background of the PCR assay and diminish below significance level.

**FIGURE 6:**
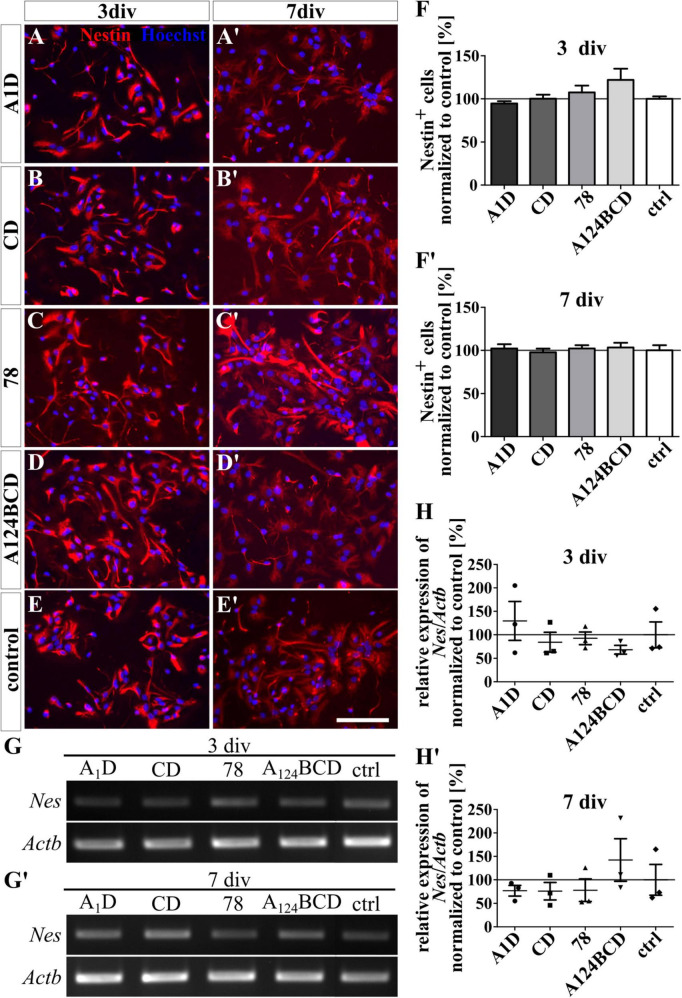
Differentiation assay of progenitor cells cultured on coated FnIII domains of Tnc. **(A–E’)** Exemplary images of the ICC for nestin-positive progenitor cells (red). Nuclei were stained with Hoechst (blue). **(F,F’)** Quantitative evaluation of the percentage of nestin-positive cells normalized to the control. The mean of the control was set as a baseline of 100%. **(G,G’)** Exemplary images of the PCR for *Nes* and *Actb*. **(H,H’)** Evaluation of the relative expression of the gene *Nes* in relation to the expression of *Actb*. The values were normalized to the control which was set as a baseline of 100%. [scale bar: 100 μm; div = days *in vitro*; mean ± SEM; ICC: *N* = 3, *n* = 18; **(F’)** ANOVA with *post-hoc* Bonferroni’s test, **(F)** Kruskal–Wallis test with *post-hoc* Dunn’s test; PCR: *N* = 3, *n* = 3, **(H,H’)** Kruskal–Wallis test with *post-hoc* Dunn’s test].

**FIGURE 7:**
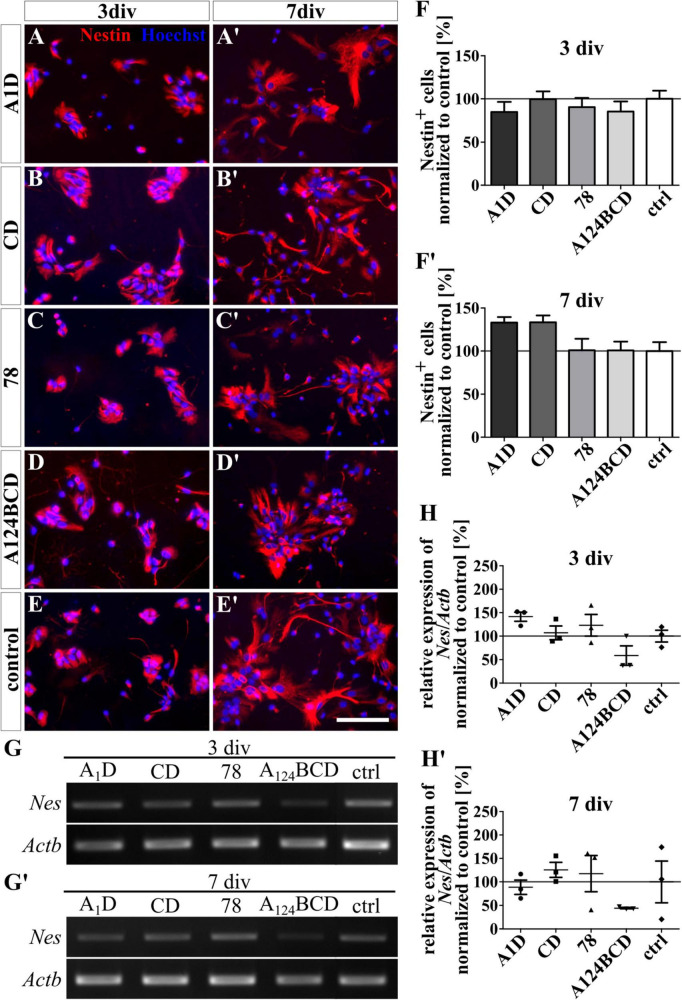
Differentiation assay of progenitor cells treated with soluble FnIII domains of Tnc. **(A–E’)** Exemplary images of the ICC for nestin-positive progenitor cells (red). Nuclei were stained with Hoechst (blue). **(F–F’)** Quantitative evaluation of the percentage of nestin-positive cells normalized to the control. The mean of the control was set as a baseline of 100%. **(G,G’)** Exemplary images of the PCR for *Nes* and *Actb*. **(H,H’)** Evaluation of the relative expression of the gene *Nes* in relation to the expression of *Actb*. The values were normalized to the control which was set as a baseline of 100%. [scale bar: 100 μm; div = days *in vitro*; mean ± SEM; ICC: *N* = 3, *n* = 18; **(F,F)** Kruskal–Wallis test with *post-hoc* Dunn’s test; PCR: *N* = 3, *n* = 3; **(H,H’)** Kruskal–Wallis test with *post-hoc* Dunn’s test].

The tested FnIII domains seemed to have no effect on the self-sustainability of nestin-positive progenitor cells, neither as a coating (see Figures 6A–F’) nor as a soluble additive (see Figures 7A–F’ and see values in Supplementary Table 4). This was confirmed by the relative expression of the marker gene for progenitor cells *Nes* via PCR in both groups, the coated cultures (see Figures 6G–H’) and the cultures treated with the soluble additives (see Figures 7G–H’ and see values in Supplementary Table 5).

### 3.3 The soluble FnIII domain 78 promoted cell divisions under proliferation conditions compared to the alternatively spliced domain A124BCD

**FIGURE 8:**
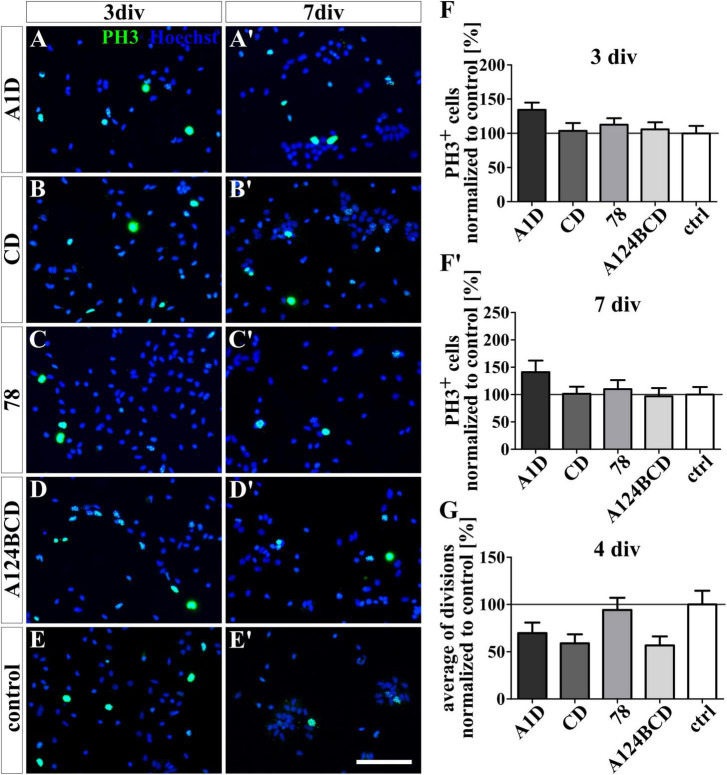
Proliferation assay of NSPCs cultured on coated FnIII domains of Tnc. **(A-E’)** Exemplary images of the ICC for PH3-positive progenitor cells (green) under differentiation condition. Nuclei were stained with Hoechst (blue). **(F,F’)** Quantitative evaluation of the percentage of PH3-positive cells under differentiation conditions normalized to the control. The mean of the control was set as a baseline of 100%. **(G)** Quantification of the average of cell divisions tracked with a video microscope under proliferation condition for 4 div. The values were normalized to the control which was set as a baseline of 100%. [scale bar: 100 μm; div = days *in vitro*; mean ± SEM; ICC: *N* = 3, *n* = 18; **(F’)** ANOVA with *post-hoc* Bonferroni’s test, **(F)** Kruskal–Wallis test with *post-hoc* Dunn’s test; video microscope: *N* = 3, *n* = see chapter 2.7; **(G)** Kruskal–Wallis test with *post-hoc* Dunn’s test].

**FIGURE 9:**
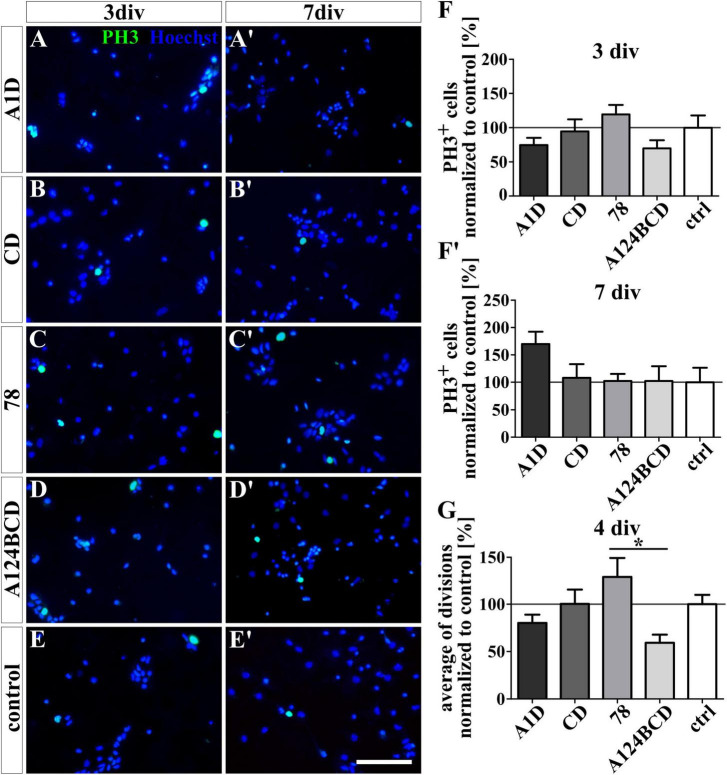
Proliferation assay of NSPCs treated with soluble FnIII domains of Tnc. **(A-E’)** Exemplary images of the ICC for PH3-positive progenitor cells (green) under differentiation condition. Nuclei were stained with Hoechst (blue). **(F,F’)** Quantitative evaluation of the percentage of PH3-positive cells under differentiation conditions normalized to the control. The mean of the control was set as a baseline of 100%. **(G)** Quantification of the average of cell divisions tracked with a video microscope under proliferation condition for 4 div. The values were normalized to the control which was set as a baseline of 100%. [scale bar: 100 μm; div = days *in vitro*; mean ± SEM; ICC: *N* = 3, *n* = 18; **(F,F’)** Kruskal–Wallis test with *post-hoc* Dunn’s test; video microscope: *N* = 3, *n* = see chapter 2.7; **(G)** Kruskal–Wallis test with *post-hoc* Dunn’s test; *p ≤ 0.05].

The proliferation of the NSPCs was analyzed under differentiation conditions with an ICC staining and under proliferation conditions with video microscopy. The staining of PH3 which is present during M-phase revealed no significant differences between the number of proliferating cells within the cultures treated with the FnIII domains as a coating (see Figures 8A–F’) or as a soluble additive (see Figures 9A–F’ and see values in Supplementary Table 4) after 3 and 7 div under differentiation conditions. However, under proliferation conditions the cells were tracked with the video microscope and every visible cell division was counted. The number of divisions was divided by the number of examined cells to achieve the average of cell divisions for every condition. It must be noticed that the cells tended to form sphere-like formation within which the cell divisions were not detectable anymore. An increased average of cell divisions was observed in the cultures treated with the soluble domain 78 compared to the soluble domain A124BCD (s78 129.3 ± 20% vs. sA124BCD 59.2 ± 8.6%, *p* ≤ 0.05; see Figure 9G) within 4 div. When considering the unnormalized values, it was seen that the cells treated with the soluble FnIII domain 78 (0.91 divisions/cell ± 0.14 divisions/cell) divided more than twice as often as the cells treated with the soluble FnIII domain A124BCD (0.42 divisions/cell ± 0.06 divisions/cell; see values in Supplementary Table 7). The other soluble domains did not induce an altered number of cell divisions compared to the control or each other (see Figure 9G and see values in Supplementary Table 7). The cultivation of NSPCs on coated FnIII domains under proliferation conditions did not significantly impact the number of cell divisions (see Figure 8G) indicating that the mode of presentation is important for the effect of the domains.

### 3.4 The Tnc-derived FnIII domain 78 increased the migrated distance covered by NSPCs

**FIGURE 10:**
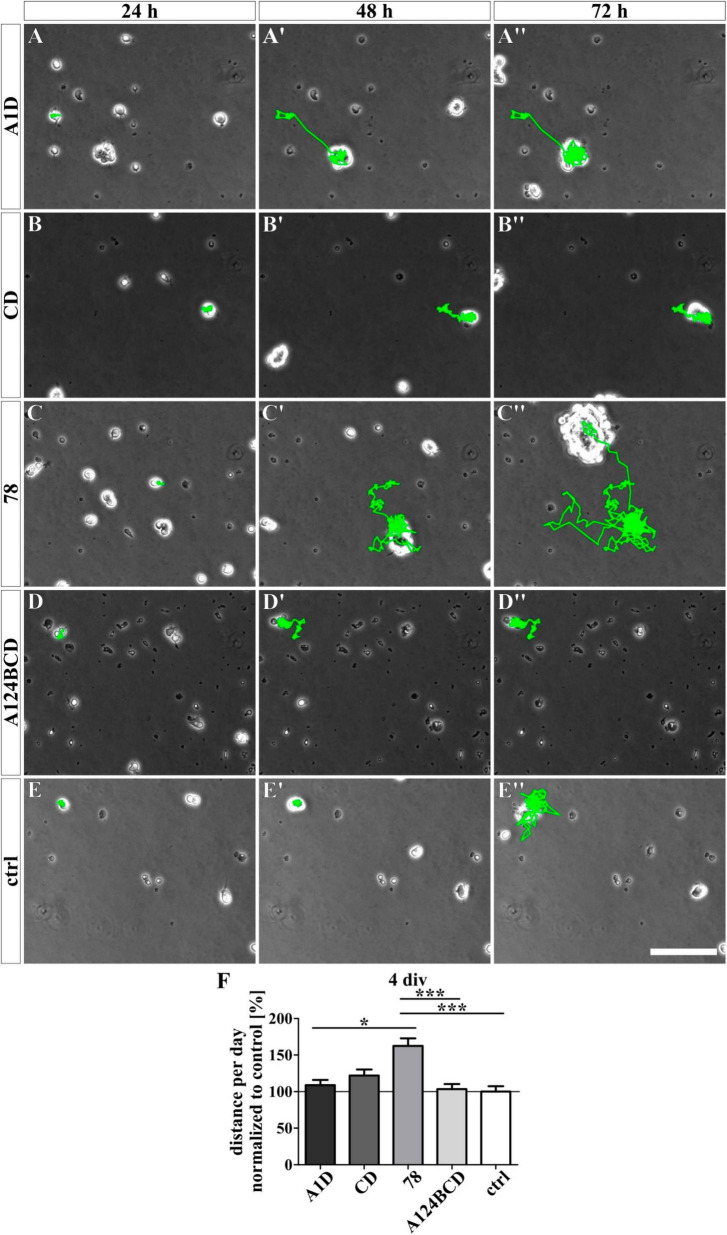
Analysis of the migration of NSPCs cultured on coated FnIII domains of Tnc for 4 div. **(A-E”)** Exemplary images of migrating NSPCs under proliferation conditions in a video microscope. Migrated distance was labeled in green. **(F)** Quantitative evaluation of the traveled distance per day by one cell normalized to the control. The mean of the control was set as a baseline of 100%. [scale bar: 100 μm; div = days *in vitro*; mean ± SEM; *N* = 3, *n* = see chapter 2.8; **(F)** Kruskal–Wallis test with *post-hoc* Dunn’s test; *p ≤ 0.05, ***p ≤ 0.001].

**FIGURE 11:**
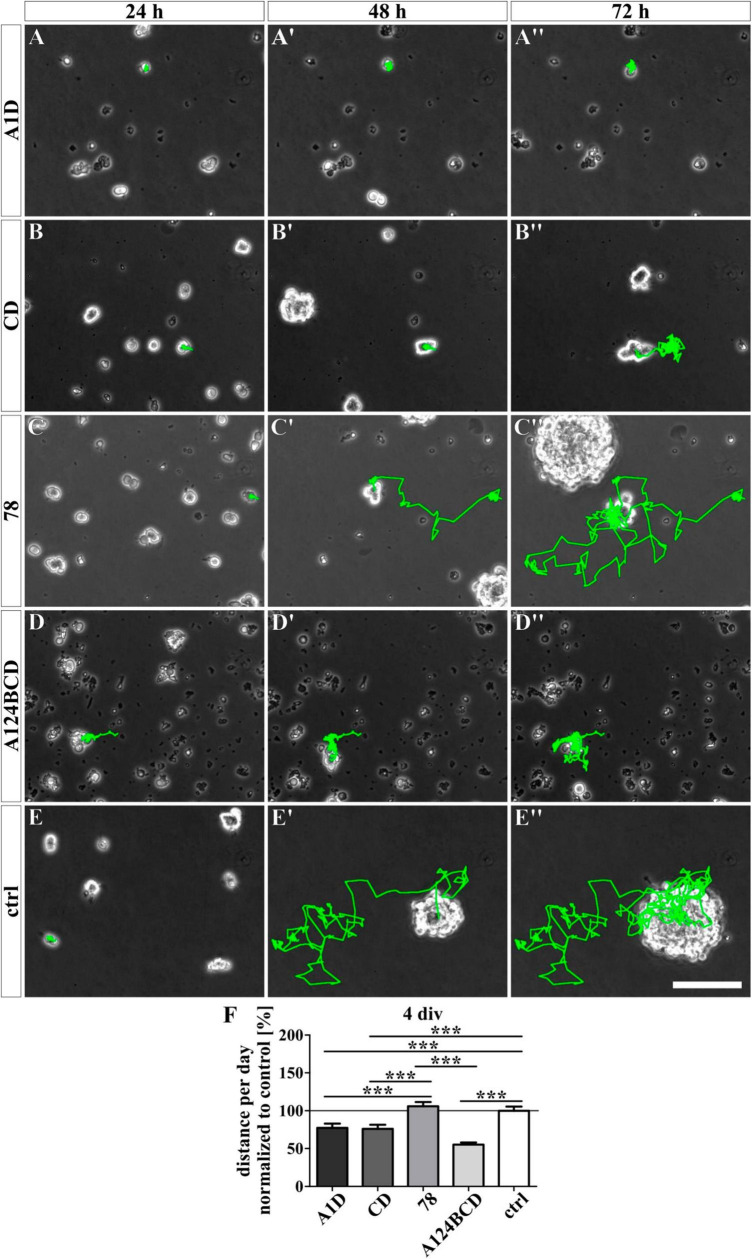
Analysis of the migration of NSPCs treated with soluble FnIII domains of Tnc for 4 div. **(A-E”)** Exemplary images of migrating NSPCs under proliferation conditions in a video microscope. Migrated distance was labeled in green. **(F)** Quantitative evaluation of the traveled distance per day by one cell normalized to the control. The mean of the control was set as a baseline of 100%. [scale bar: 100 μm; div = days *in vitro*; mean ± SEM; *N* = 3, *n* = see chapter 2.8; **(F)** Kruskal–Wallis test with *post-hoc* Dunn’s test; ***p ≤ 0.001].

For the analysis of the effect of the different Tnc-derived FnIII domains on the migratory ability of the NSPCs, the cells were tracked with a video microscope. The NSPCs cultured on the coated FnIII domain 78 migrated a significantly longer distance per day compared to the cells on the coated FnIII domains A1D (c78 162.6 ± 10.4% vs. cA1D 108.9 ± 7.2%, *p* ≤ 0.05) and A124BCD (c78 162.6 ± 10.4% vs. cA124BCD 103.3 ± 7.1%, *p* ≤ 0.001) as well as a 62.6% longer distance per day than the cells in the control (c78 162.6 ± 10.4% vs. control 100 ± 7.4%, *p* ≤ 0.001; see Figures 10A–F and Supplementary Videos 1–5). The cells treated with the coated FnIII 78 domain gained a speed of 17 ± 1.1 μm/h (see values in Supplementary Table 7). The treatment with the soluble domains A1D, CD and A124BCD reduced the migrated distance of the NSPCs per day compared to the control by 22.5, 23.9, and 44.8%, respectively (sA1D 77.5 ± 5.5% vs. control 100 ± 5.5%, *p* ≤ 0.001; sCD 76.1 ± 5.4% vs. control 100 ± 5.5%, *p* ≤ 0.001; sA124BCD 55.2 ± 2.8% vs. control 100 ± 5.5%, *p* ≤ 0.001; see Figures 11A–F and Supplementary Videos 6–10). In contrast, the soluble FnIII domain 78 promoted significantly the migratory ability of the NSPCs in comparison to the soluble domains A1D, CD and A124BCD by 36.8, 39.3, and 92%, respectively (s78 106 ± 5.5% vs. sA1D 77.5 ± 5.5%, *p* ≤ 0.001; s78 106 ± 5.5% vs. sCD 76.1 ± 5.4%, *p* ≤ 0.001; s78 106 ± 5.5% vs. sA124BCD 55.2 ± 2.8%, *p* ≤ 0.001). The cells treated with the soluble FnIII domain 78 (22.3 ± 1.2 μm/h) migrated nearly twice as fast as the cells treated with the domain A124BCD (11.6 ± 0.6 μm/h; see values in Supplementary Table 7). Furthermore, the cells treated with the soluble domain 78 and the cells in the control migrated nearly the same distance per day (see Figures 11C–F), indicating that the constitutively expressed domain did not cause the migration inhibiting effect that the alternatively spliced domains elicited. In summary, the domains of the alternatively spliced region possessed a migration inhibiting effect, while the domain 78 of the constitutively expressed region exhibited a migration stimulating effect.

### 3.5 The Tnc-derived FnIII domains did not activate the intracellular signaling pathways which include the proteins notch, Erk, Akt, and FAK

**FIGURE 12:**
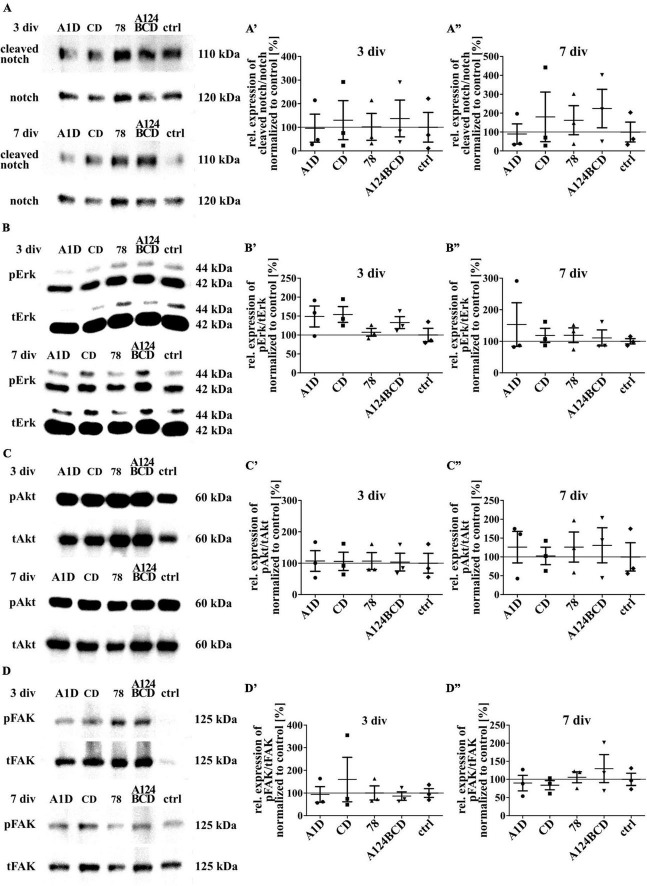
Analysis of the activation of intracellular signaling pathways including the proteins notch, Erk, Akt and FAK in NSPCs cultured on coated FnIII domains of Tnc. **(A–D)** Exemplary images of western blots using antibodies against cleaved notch, notch, pErk, tErk, pAkt, tAkt, pFAK and tFAK. **(A’–D”)** Quantification of the relative expression of the phosphorylated or cleaved proteins in relation to the total proteins. The values were normalized to the control. The mean of the control was set as a baseline of 100%. (div = days *in vitro*; mean ± SEM; *N* = 3, *n* = 3; Kruskal–Wallis test with *post-hoc* Dunn’s test).

**FIGURE 13:**
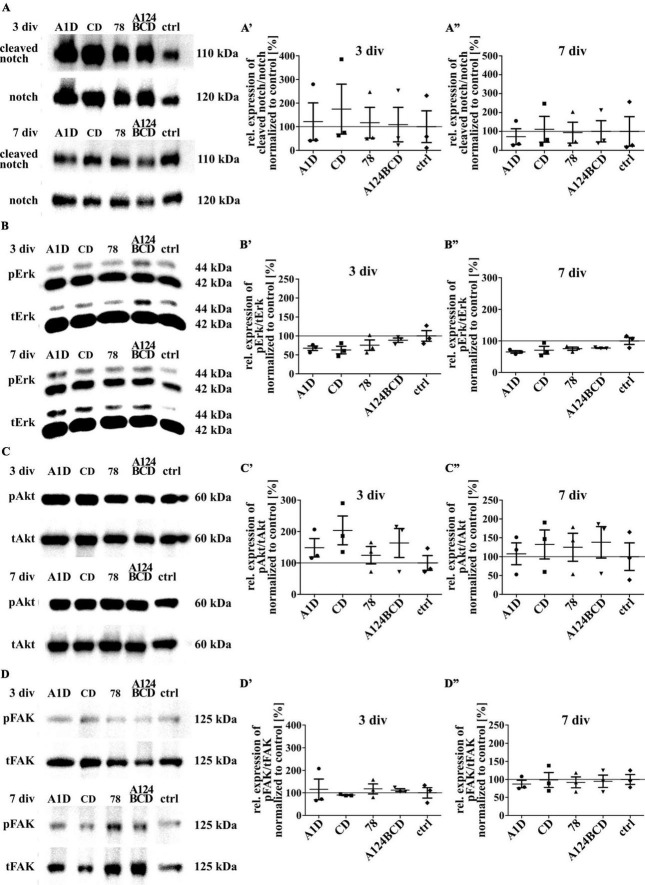
Analysis of the activation of intracellular signaling pathways including the proteins notch, Erk, Akt and FAK in NSPCs treated with soluble FnIII domains of Tnc. **(A–D)** Exemplary images of western blots using antibodies against cleaved notch, notch, pErk, tErk, pAkt, tAkt, pFAK, and tFAK. **(A’–D”)** Quantification of the relative expression of the phosphorylated or cleaved proteins in relation to the total proteins. The values were normalized to the control. The mean of the control was set as a baseline of 100%. (div = days *in vitro*; mean ± SEM; *N* = 3, *n* = 3; Kruskal–Wallis test with *post-hoc* Dunn’s test).

Tenascin-C is able to activate signaling pathways directly by binding to integrins or the EGF-receptor and indirectly by binding to co-receptors as well as by the interaction with extracellular components such as growth factors or morphogens (Faissner et al., 2017). To investigate the reasons for the previously described effects the activation of some intracellular signaling pathways was investigated by SDS-PAGE and western blot analysis. The protein notch promotes a proliferative signal during neurogenesis and is cleaved by activation. It is also important for the determination of the cell fate, differentiation and cell death (Bray, 2006; Kopan, 2012). The comparison of the relation of cleaved notch signal to total notch signal did not differ between the different domains within the two groups of coated (see Figures 12A–A”) and soluble treatments (see Figures 13A–A”; see values in Supplementary Table 6) after 3 and 7 div. The proteins Erk1 and Erk2 are part of the MAPK signaling pathway which regulates the cell cycle and is important for fundamental cellular processes like cell proliferation, survival, growth, metabolism, migration and differentiation (Lavoie et al., 2020). It is activated by phosphorylation. The amount of phosphorylated Erk in relation to total Erk did not differ between the different domains, neither in the coated group (see Figures 12B–B”), nor in the soluble additive group (see Figures 13B–B”; see values in Supplementary Table 6) after 3 and 7 div. The protein Akt is phosphorylated in a multi-step process that involves PI3K and is part of an intrinsic signaling pathway that regulates cell metabolism, growth, proliferation, and survival (Hemmings and Restuccia, 2012). The quantification of the intensity of the western blot signal of the phosphorylated Akt in relation to total Akt did not display significant differences between the different domains within each group, namely the coated cultures (see Figures 12C–C”) and the cultures treated with the soluble domains (see Figures 13C–C”; see values in Supplementary Table 6) after 3 and 7 div. The FAK is involved in the cellular adhesion and motility. It is phosphorylated by activation in response to integrin binding which among others interacts with Tnc (Mitra et al., 2005). The amount of phosphorylated FAK in relation to total FAK did not differ between the tested FnIII domains within the coating group (see Figures 12D–D”) and the soluble additive group (see Figures 13D–D”; see values in Supplementary Table 6) after 3 and 7 div.

### 3.6 The Tnc-derived FnIII domains did not regulate the expression of Sam68, Vav3, or Tnc itself

**FIGURE 14:**
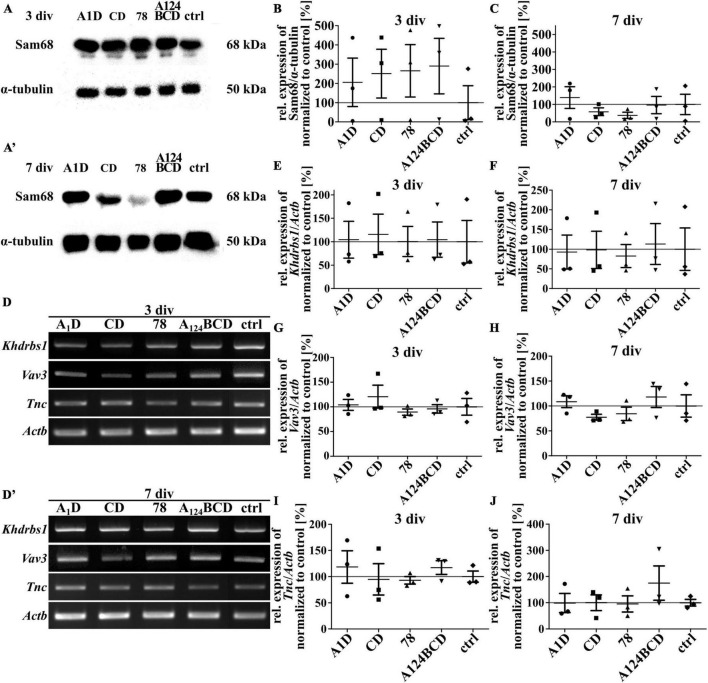
Analysis of the relative expression of Sam68/*Khdrbs1*, *Vav3*, and *Tnc* in NSPCs cultured on coated FnIII domains of Tnc. **(A–A’)** Exemplary images of western blots using antibodies against Sam68 and α-tubulin. **(B,C)** Quantification of the relative expression of Sam68 in relation to α-tubulin. The values were normalized to the control. The mean of the control was set as a baseline of 100%. **(D,D’)** Exemplary images of the PCR for the genes *Khdrbs1*, *Vav3*, *Tnc*, and *Actb*. **(E–J)** Evaluation of the relative expression of the genes *Khdrbs1*, *Vav3*, and *Tnc* in relation to the expression of *Actb*. The values were normalized to the control which was set as a baseline of 100%. (div = days *in vitro*; mean ± SEM; *N* = 3, *n* = 3; Kruskal–Wallis test with *post-hoc* Dunn’s test).

**FIGURE 15:**
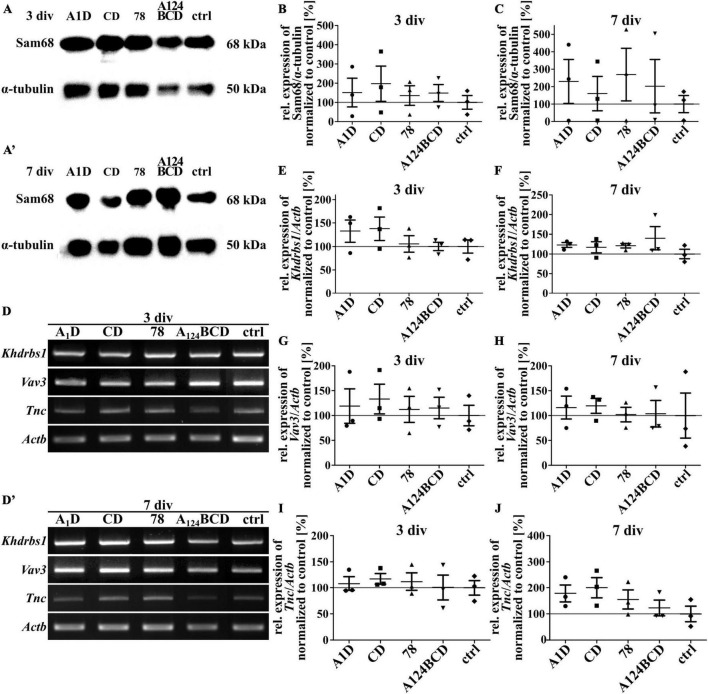
Analysis of the relative expression of Sam68/*Khdrbs1*, *Vav3*, and *Tnc* in NSPCs treated with soluble FnIII domains of Tnc. **(A,A’)** Exemplary images of western blots using antibodies against Sam68 and α-tubulin. **(B,C)** Quantification of the relative expression of Sam68 in relation to α-tubulin. The values were normalized to the control. The mean of the control was set as a baseline of 100%. **(D,D’)** Exemplary images of the PCR for the genes *Khdrbs1*, *Vav3*, *Tnc*, and *Actb*. **(E–J)** Evaluation of the relative expression of the genes *Khdrbs1*, *Vav3*, and *Tnc* in relation to the expression of *Actb*. The values were normalized to the control which was set as a baseline of 100%. (div = days *in vitro*; mean ± SEM; *N* = 3, *n* = 3; Kruskal–Wallis test with *post-hoc* Dunn’s test).

Sam68 is a RNA-binding protein and is involved in several steps of mRNA processing, like transcription and alternative splicing as well as in regulation of cellular processes like signal transduction, cell cycle regulation and tumorigenesis (Lukong and Richard, 2003; Bielli et al., 2011). An overexpression of Sam68 favored the production of large Tnc isoforms (Moritz et al., 2008). The guanine nucleotide exchange factor (GEF) Vav3 activates several members of the Rho GTPase family and thus has a regulatory influence on the rearrangement of the cytoskeleton (Movilla and Bustelo, 1999). It was published that Tnc induced the downregulation of Sam68 and Vav3 in NSPCs cultures (Moritz et al., 2008). To investigate whether one of the tested FnIII domains was responsible for the downregulation of these proteins the relative expression of them was analyzed on mRNA and protein level. When comparing the protein level of Sam68 in cultures treated with the coated (see Figures 14A, A’, B, C) and soluble FnIII domains (see Figures 15A, A’, B, C; see values in Supplementary Table 6) no differences were found within the groups. This result was confirmed by the analysis of the relative expression of *Khdrbs1* (KH RNA binding domain containing, signal transduction associated 1; gene for Sam68) on mRNA level of the coated cultures (see Figures 14D, D’, E, F) and the cultures treated with the soluble additives (see Figures 15D, D’, E, F; see values in Supplementary Table 5). The relative expression of *Vav3* was analyzed on mRNA level. The quantification revealed that the expression of *Vav3* was not altered after the cultivation of NSPCs on coated domains (see Figures 14D, D’, G, H) or after the treatment with soluble domains (see Figures 15D, D’, G, H; see values in Supplementary Table 5) after 3 and 7 div compared to the control. To find out whether the tested FnIII domains of Tnc had an effect on the expression of Tnc itself, the relative expression of *Tnc* was analyzed on mRNA level. The cells which were cultured on the coated FnIII domains did not exhibit an altered expression of *Tnc* compared to the control after 3 and 7 div (see Figures 14D, D’, I, J). The same results were observable in the cultures treated with the soluble FnIII domains (see Figures 15D, D’, I, J; see values in Supplementary Table 5). In summary, the results revealed that the tested FnIII domains of Tnc, namely A1D, CD, 78 and A124BCD, did not have an influence on the expression of Sam68/*Khdrbs1*, *Vav3*, or *Tnc* itself.

## 4 Discussion

In the present study, we analyzed the effect of the alternatively spliced FnIII domain combinations A1D, CD, A124BCD as well as of the constitutively expressed domain 78 of Tnc on the differentiation, proliferation and migration of NSPCs as well as the activation and expression of signaling proteins. Additionally, the impact of the various modes of domain presentations, namely the immobilized coating and the soluble additive into the medium, on the cells was investigated.

The importance of Tnc for neural stem cells becomes obvious through its expression pattern. During development it is produced by radial glial and/or astrocyte progenitor cells (Stoykova et al., 1997; Yuasa, 2001), but its expression decreases early postnataly and is in adulthood restricted to the stem cell niches, for example to the subventricular zone of the lateral ventricle (Götz et al., 1998; Chiquet-Ehrismann et al., 2014; Faissner et al., 2017; Kjell et al., 2020). During the process of downregulation, a shift from large to small isoforms has been recognized (Bartsch et al., 1992; Dörries and Schachner, 1994). Due to the great number of isoforms, it is assumed that every variant may have a different effect on NSPCs (Joester and Faissner, 1999, 2001; von Holst et al., 2007; Theocharidis et al., 2012).

The functions of the single FnIII domains of Tnc were mostly analyzed regarding their effects on neurite outgrowth. Thereby, the analysis of different types of neurons reveals an increased neurite outgrowth mediated by the domains FnIII BD, D6, and 6 (Husmann et al., 1992; Götz et al., 1996; Rigato et al., 2002; Michele and Faissner, 2009). The FnIII domain BD-induced neurite outgrowth might be due to the interaction with contactin and the β1-integrin subunit which might activate a phospholipase C-, protein kinase C- and calmodulin kinase-dependent pathway (Rigato et al., 2002; Michele and Faissner, 2009). Within the FnIII D domain the peptide sequence VFDNFVLK has turned out to promote neurite outgrowth (Meiners et al., 2001). Within this sequence two amino acid pairs, namely FD and FV, are found to be required for this activity and are crucial for the interaction with α7β1 integrin (Mercado et al., 2004). Another peptide motif, namely VSWRAPTA within the FNIII 6 domain, promotes the outgrowth of neurites by the activation of the FAK and the Erk 1/2 pathway (Jarocki et al., 2019). In the present study, the tested domains containing the neurite outgrowth-supporting FnIII domain D, namely in FnIII A1D and CD, did not promote the differentiation of neurons, indicating that the above-mentioned effects triggered selectively neurons but not NSPCs. In fact, the activation of the aforementioned tested signaling pathways FAK and Erk was not altered. However, in this study, we saw an increased differentiation of neurons cultured with the soluble FnIII domain A124BCD compared to the control, indicating that a combination of several domains is necessary for the differentiation of neurons. However, in this domain combination the domains B and D were included, suggesting that this composition might have an effect on NSPCs. Taken together, these results fit with the statement that in early developmental phases during neurogenesis the long Tnc isoforms are preferentially expressed.

Additionally, we saw an effect in the astrocyte differentiation caused by the FnIII domain combination A1D. When examining the glial scar, Bijelić et al. (2021) reported that the FnIII domain D and its combination with the FnIII domain A are most supportive. The authors refer an increased GFAP immunoreactivity in Tnc wild-type astrocytes treated with FnIII domain D and with a combination of FnIII domain D and A compared to treated Tnc knockout astrocytes. This supports our results regarding an increased number of GFAP-positive astrocytes in Tnc wild-type cultures treated with the FnIII A1D domain. This points to an astrocyte production- and astrocyte reactivity-supporting effect of the FnIII domains A1D which might also be associated with glial scar formation. In fact, FnIII domain D is upregulated in brain injuries at 2–4 days after lesion (Dobbertin et al., 2010).

Previous studies regarding the effects of Tnc on oligodendrocytes are related to migration and maturation of oligodendrocyte precursor cells (OPCs). Thereby, an adhesion repulsive effect of the alternatively spliced FnIII domains on OPCs are detected, while the migration of the cells is inhibited by the FnIII 78 domain (Kiernan et al., 1996). It could be shown that the migration capability of OPCs on Tnc coating is dependent on the GEF Vav3 (Schäfer et al., 2022). Furthermore, the maturation of OPCs is inhibited by Tnc substrate, as seen in a reduced myelin basic protein (MBP) expression which might be related to an interfering with Akt phosphorylation and contactin binding (Czopka et al., 2009, 2010). Moreover, Tnc has a negative effect on myelination and remyelination of oligodendrocytes (Bauch and Faissner, 2022). However, little is known about the influence of the single FnIII domains of Tnc on the differentiation of oligodendrocytes from NSPCs. We found an increased oligodendrocyte population in cultures treated with the soluble A124BCD domain compared to the control as well as to the treatment with the soluble domains A1D and 78, indicating that the large Tnc isoform promoted the differentiation of oligodendrocytes in a 3D environment but not as a substrate. Differentiation of oligodendrocytes occurs in waves. The first two waves appear at E12.5 and E14.5 (Michalski and Kothary, 2015). During this time point the large isoform of Tnc is dominantly expressed (Bartsch et al., 1992; Dörries and Schachner, 1994) and might support the differentiation of oligodendrocytes from NSPCs. The third wave of oligodendrocytes generation happens postnatally (Michalski and Kothary, 2015) when Tnc expression is downregulated (Bartsch et al., 1992; Dörries and Schachner, 1994). This indicates that the alternatively spliced area of Tnc might have an influence on the differentiation of oligodendrocytes of the first two waves but not on the third. However, neither an increased activation of Akt, nor an altered expression of Vav3 was detectable, whose participation in the interplay between Tnc and OPCs had been shown before. A contribution of these molecules in the observed effect cannot be excluded due to the analysis of a mixed culture rather than of purified differentiated oligodendrocytes.

Unfortunately, the analysis of the signaling pathways and gene expression did not result in significant findings. Due to the analysis of a mixed culture with different cell types, the differences in a given lineage between control and treated populations represented a minor fraction of the overall culture. Furthermore, several signaling pathways are shared between the different lineages. This situation in conjunction with inter experimental variability probably entailed that the western blot and PCR results of the intracellular effects of the individual cell types became blurred and failed to achieve statistical significance.

The influence of Tnc on the migration is thoroughly analyzed in tumors like glioblastoma and is supposed to induce a highly agile and invasive phenotype (Deryugina and Bourdon, 1996; Hirata et al., 2009; Brösicke and Faissner, 2015). In contrast, an anti-migratory effect of Tnc is observed in adult neuroblasts derived from wild-type mice compared to neuroblasts from Tnc knockout mice which migrate in significantly longer trajectories (Schaberg et al., 2022). However, it could be shown that blocking of different FnIII domains of Tnc inhibits the migration of granule cells in cerebellar explants (Husmann et al., 1992), indicating that migratory or anti-migratory effect of Tnc might be domain- as well as cell type-specific. For example, the FnIII domain A1A2A4 turns out to be anti-adhesive and promotes the migration and repulsion of neurons, whereas the same domain supports the adhesion of tumor cell lines via the interaction with RPTPβ (Götz et al., 1996; Adamsky et al., 2001). In contrast, the coated domain A124BD supports the attachment of neurons (Götz et al., 1996). Additionally, the domains FnIII A and D decrease the migration of astrocytes in a scratch wound assay (Bijelić et al., 2021). Furthermore, it has been shown that the alternatively spliced FnIII domains from A-D bind with a high affinity to the cell surface receptor annexin II (Chung and Erickson, 1994). This receptor is known to inhibit the migration of the cells (Balch and Dedman, 1997). In the present study, an increased migratory ability was found with the coated 78 domain which is not included in the alternatively spliced region. In contrast, the soluble domains of the alternatively spliced segment displayed a decreased migratory ability compared to the constitutively expressed domain and the control. The binding of the surface annexin II receptor and RPTPβ might be an explanation for the decreased migration induced by the alternatively spliced domains.

As a member of the neural stem cell niche Tnc influences the proliferation of NSPC (Faissner et al., 2017). This has been seen on the one hand, in a reduced rate of cell divisions and a shortened cell cycle length in embryonic and adult Tnc knockout NSPCs of murine origin and on the other hand, in an enhanced proliferation during gliogenesis in the spinal cord of Tnc knockout mice (Karus et al., 2011; May et al., 2018; Schaberg et al., 2022). It has been shown that Tnc promotes proliferation via its receptor αvβ3 integrin in OPCs and in paired box 6 (Pax6)-negative, T-box brain protein 2 (Tbr2)-positive intermediate progenitors (Garcion et al., 2001; Stenzel et al., 2014). Another receptor that, among others, regulates cell proliferation is the EGF-receptor which can be activated by the EGF-like repeats of Tnc, while the absence of Tnc results in a delayed expression of the EGF-receptor (Swindle et al., 2001; Garcion et al., 2004; Iyer et al., 2007; Wee and Wang, 2017). Although it has been shown before that Tnc can modulate the proliferation of NSPCs in different ways, the relation of the alternatively spliced domains has not been examined before. Our results displayed that the cell division rate was decreased by the soluble domains that include the total alternatively spliced region in comparison to the soluble FnIII domain 78 which is part of the constitutively expressed domains. In fact, the proliferation-promoting integrin αvβ3 does not bind to the alternatively spliced segment (Joester and Faissner, 2001). This indicates that different isoforms of Tnc might cause the aforementioned contrary effects on the proliferation of NSPCs.

In this study, some domain combinations had an influence on the behavior of NSPCs but remarkably this effect was mostly visible only in one mode of treatment but not in both. Different effects with substrate-bound and soluble total Tnc have been seen before regarding neurite outgrowth (Lochter et al., 1991). This dichotomy in the effect of coated and soluble Tnc is still unclear but might be related to the difference in presentation (Lochter et al., 1991). The authors suggested that different domains are responsible for the promotion of the neurite outgrowth and its inhibition. Thus, the mode of presentation might hide or present different domains that mediate a specific effect. Furthermore, soluble domains are more flexible which might generate more temporary bindings, whereas coated domains are immobilized and more permanent. The time period of binding might be crucial for its effect (van der Velden et al., 2020). Recently, it could be shown that covalent, irreversible interactions between cells and the matrix have a different effect on cells than non-covalent, temporary bindings (Liu et al., 2023).

The use of single domains instead of the total protein has many advantages for regenerative medicine due to controllable effects. In order to create the best possible natural environment *in vitro*, hydrogels were often used (Caliari and Burdick, 2016; Glotzbach et al., 2020). These matrices can be tailored regarding stiffness, charge and biodegradability and can thereby mimic the *in vivo* setting of the cells (Huebsch et al., 2010; Wade and Burdick, 2012; Stamm et al., 2022). Many hydrogels consist of natural products like Matrigel or of ECM components like collagen, elastin, fibrin and gelatin (Hughes et al., 2010; Catoira et al., 2019). Although these hydrogels have many advantages, the ingredients which are mostly ECM molecules with specific characteristics and effects guide the cells already in a certain direction and do not act as a neutral basis. In contrast, xeno-free hydrogels consisting of artificial ingredients represent an impartial foundation. This scaffold can be modified with single domains or peptides whose properties can be analyzed independently from the background (Lee et al., 2010; Jang et al., 2013; Sallouh et al., 2017). Furthermore, cell fates can be triggered by domains whose properties are already known. The domains of Tnc might be promising candidates to derivatize such hydrogels. Cultures of NSPCs in hydrogels containing the A124BCD domain might trigger the differentiation of neurons and oligodendrocytes, whereas A1D-modified hydrogels might support the differentiation of astrocytes. The proliferation and migration of NSPCs might be promoted by hydrogels modified with the FnIII domain 78.

## 5 Conclusion

In this study, we showed the effects of the Tnc-derived FnIII domains A1D, CD, A124BCD, and 78 on NSPCs. While the A124BCD domain supported the differentiation of neurons and oligodendrocytes, the FnIII domain A1D might have a astrocyte-stimulating effect. The FnIII domain 78 had a positive impact on the proliferation and migration of NSPCs. Until now the underlying mechanisms causing these effects have not yet been identified. However, in further studies a closer look on the single destinations of cell fates would be necessary including continuing analysis of signaling pathways. Taken together, the outcome of these studies might be used in regenerative medicine as a tool to modulate NSPCs behavior. A combination of biologically active ECM fragments with 3D cultures, for example artificial hydrogels loaded with Tnc domains, might represent a promising instrument for the future of transplantation research.

## Data availability statement

The original contributions presented in the study are included in the article/Supplementary material, further inquiries can be directed to the corresponding author.

## Ethics statement

The animal study was approved by the Landesamt für Natur, Umwelt und Verbraucherschutz (LANUV) Nordrhein-Westfalen. The study was conducted in accordance with the local legislation and institutional requirements.

## Author contributions

KG: Investigation, Writing – original draft, Validation, Visualization. AF: Funding acquisition, Writing – review and editing, Project administration, Supervision.
