# Supplementary material for: Substrate-bound and soluble domains of tenascin-C regulate differentiation, proliferation and migration of neural stem and progenitor cells
Source: Front Cell Neurosci. 2024 Feb 14;18:1357499. doi: 10.3389/fncel.2024.1357499 (PMC10902920; doi:10.3389/fncel.2024.1357499)
Supplement: Supplementary file 1 [file Data_Sheet_1.pdf]

frontiers

Supplementary Material

Substrate-bound and soluble Domains of Tenascin-C regulate Differentiation, Proliferation and Migration of Neural Stem and Progenitor Cells

Kristin Glotzbach, Andreas Faissner*

*** Correspondence:** Andreas Faissner: andreas.faissner@rub.de

Supplementary Video 1: FnIII_A1D_coated. Exemplary video of the migration of NSPCs cultured on coated Tnc-derived FnIII domain A1D under proliferation conditions in a video microscope for 4 days. Migrated distance was labeled in green.

Supplementary Video 2: FnIII_CD_coated. Exemplary video of the migration of NSPCs cultured on coated Tnc-derived FnIII domain CD under proliferation conditions in a video microscope for 4 days. Migrated distance was labeled in green.

Supplementary Video 3: FnIII_78_coated. Exemplary video of the migration of NSPCs cultured on coated Tnc-derived FnIII domain 78 under proliferation conditions in a video microscope for 4 days. Migrated distance was labeled in green.

Supplementary Video 4: FnIII_A124BCD_coated. Exemplary video of the migration of NSPCs cultured on coated Tnc-derived FnIII domain A124BCD under proliferation conditions in a video microscope for 4 days. Migrated distance was labeled in green.

Supplementary Video 5: Control_coated. Exemplary video of the migration of NSPCs cultured on poly-L-ornithine substrate without further treatment under proliferation conditions in a video microscope for 4 days. Migrated distance was labeled in green.

Supplementary Video 6: FnIII_A1D_soluble. Exemplary video of the migration of NSPCs cultured on poly-L-ornithine substrate treated with soluble Tnc-derived FnIII domain A1D under proliferation conditions in a video microscope for 4 days. Migrated distance was labeled in green.

Supplementary Video 7: FnIII_CD_soluble. Exemplary video of the migration of NSPCs cultured on poly-L-ornithine substrate treated with soluble Tnc-derived FnIII domain CD under proliferation conditions in a video microscope for 4 days. Migrated distance was labeled in green.

Supplementary Video 8: FnIII_78_soluble. Exemplary video of the migration of NSPCs cultured on poly-L-ornithine substrate treated with soluble Tnc-derived FnIII domain 78 under proliferation conditions in a video microscope for 4 days. Migrated distance was labeled in green.

Supplementary Video 9: FnIII_A124BCD_soluble. Exemplary video of the migration of NSPCs cultured on poly-L-ornithine substrate treated with soluble Tnc-derived FnIII domain A124BCD under proliferation conditions in a video microscope for 4 days. Migrated distance was labeled in green.

Supplementary Video 10: Control_soluble. Exemplary video of the migration of NSPCs cultured on poly-L-ornithine substrate treated with PBS under proliferation conditions in a video microscope for 4 days. Migrated distance was labeled in green.

Supplementary Table 1: Collection of used primary antibodies.

Antibody	method	dilution	origin	vendor	Catalog number	RRID
O4	ICC	1:50	mouse	hybridoma clone 81 (Sommer and Schachner, 1981)		
anti-GFAP	ICC	1:300	rabbit	Agilent	Z0334	AB_10013382
anti-βIII-tubulin	ICC	1:300	mouse	Sigma-Aldrich	T8660	AB_477590
anti-PH3	ICC	1:100	rabbit	Millipore	06-570	AB_310177
anti-nestin	ICC	1:500	mouse	Millipore	MAB353	AB_94911
anti-pErk	western blot	1:1000	rabbit	Cell Signaling Technology	9101	AB_331646
anti-tErk	western blot	1:1000	mouse	Santa Cruz Biotechnology	sc-271269	AB_10611091
anti-pAkt	western blot	1:2000	rabbit	Cell Signaling Technology	4060	AB_2315049
anti-tAkt	western blot	1:1000	rabbit	Cell Signaling Technology	4691	AB_915783
anti-pFAK	western blot	1:1000	rabbit	Cell Signaling Technology	3283	AB_2173659
anti-tFAK	western blot	1:1000	rabbit	Cell Signaling Technology	3285	AB_2269034
anti-notch	western blot	1:1000	rabbit	Cell Signaling Technology	3608	AB_2153354
anti-cleaved notch	western blot	1:1000	rabbit	Cell Signaling Technology	4147	AB_2153348
anti-Sam68	western blot	1:500	mouse	Santa Cruz Biotechnology	sc-1238	AB_627858
anti-α-tubulin	western blot	1:10000	mouse	Sigma-Aldrich	T9026	AB_477593

Supplementary Table 2: Collection of used secondary antibodies.

Antibody	method	dilution	origin	coupling	vendor	Catalog number	RRID
anti-human IgG Fc antibody	western blot dot blot	1:7000	goat	HRP-coupled	Sigma-Aldrich	A0170	AB_257868
anti-rabbit	ICC	1:300	goat	Cy2-coupled	Jackson ImmunoResearch Labs	111-545-045	AB_2338049
anti-mouse	ICC	1:300	goat	Cy3-coupled	Jackson ImmunoResearch Labs	115-165-068	AB_2338686
anti-mouse	western blot	1:5000 (1:10000 for α-tubulin)	goat	HRP-coupled	Jackson ImmunoResearch Labs	115-035-068	AB_2338505
anti-rabbit	western blot	1:5000	goat	HRP-coupled	Jackson ImmunoResearch Labs	111-035-144	AB_2307391

Supplementary Table 3: Primer sequences

Gene	Primer sequence	Annealing temperature	Cycles	GenBank accession number
Actb	For: 5'-TATGCCAACACAGTGCTGTCTGGTGG-3'<br>Rev: 5'-TAGAAGCATTTGCGGTGGACAATGG-3'	60°C	25	NM_007393.5
Tubb3	For: 5'- CATGGACAGTGTTCGGTCTG-3'<br>Rev: 5'-TGTCGATGCAGTAGGTCTC-3'	60°C	32	NM_023279.3
Gfap	For: 5'-CGACTATCGCCGCCAACTGC-3'<br>Rev: 5'-GCGATCTCGATGTCCAGGGCT-3'	60°C	32	NM_001131020.1
Pdgfra	For: 5'-GCACCAAGTCAGGTCCCATT-3'<br>Rec: 5'-CTTCACTGGTGGCATGGTCA-3'	60°C	32	NM_001083316.2
Nes	For: 5'-CTCGAGCAGGAAGTGGTAGG-3'<br>Rev: 5'-GTTAGCGCTGCCRCRAGACC-3'	60°C	32	NM_016701.3

Tnc	For: 5’-GCTCTAGATGCGGCTGTGTCTGTGAAC-3’<br>Rev: 5’-CGGGATCCGACACCGCTTTTCACTGC-3’	60°C	32	NM_001369211.1
Khdrbs1	For: 5’-ACATGGGGAGCTCCAAGATT-3’<br>Rev: 5’-TGGCACTACTGCCGTTGTTA-3’	60°C	56	NM_011317.4
Vav3	For: 5’-CCAGTGTTGACCCTGCACTA-3’<br>Rev: 5’-CTCTTCATGCTGGGGAAAAG-3’	56°C	35	NM_001378987.1

Supplementary Table 4: Collection of all evaluated and normalized values of the ICC

	**βIII-tubulin**			
	**Domain**	**Value**	**Value normalized to control**	**Statistical test**
3 div	cA1D	17.1 % ± 0.8 %	95.4 % ± 4.5 %	ANOVA with *post hoc* Bonferroni’s test
	cCD	18.2 %± 1.2 %	101.3 % ± 6.5 %	
	c78	16.4 % ± 1 %	91.1 % ± 5.4 %	
	cA124BCD	17.4 % ± 1.2 %	96.6 % ± 6.9 %	
	coated control	18 % ± 1.3 %	100 % ± 7.4 %	
	sA1D	22.8 % ± 1.9 %	136 % ± 11.1 %	ANOVA with *post hoc* Bonferroni’s test
	sCD	20.2 % ± 1.3 %	120.3 % ± 7.9 %	
	s78	19 % ± 1.3 %	113 % ± 7.7 %	
	sA124BCD	24.1 % ± 2.1 %	143.4 % ± 12.5 %	
	soluble control	16.8 % ±1.7 %	100 % ± 10.4 %	
7 div	cA1D	21.3 % ± 1.5 %	115.9 % ± 8.3 %	Kruskal-Wallis test with *post hoc* Dunn’s test
	cCD	24.6 % ± 2.2 %	133.8 % ± 11.9 %	
	c78	22.1 % ± 1.6 %	120.4 ± 8.7 %	
	cA124BCD	20.8 % ± 1.4 %	113 % ± 7.8 %	
	coated control	18.4 % ± 1.1 %	100% ± 6 %	
	sA1D	21.3 % ± 2.2 %	87.8 % ± 9.2 %	ANOVA with *post hoc* Bonferroni’s test
	sCD	21.5 % ± 2.4 %	88.8 % ± 10 %	
	s78	22.7 % ±2.5 %	93.4 % ± 10.3 %	
	sA124BCD	18.3 % ± 1.6 %	75.6 % ± 6.7 %	
	soluble control	24.3 % ± 2.5 %	100 % ± 10.2 %	
	**GFAP**			
	**Domains**	**Value**	**Value normalized to control**	**Statistical test**
3 div	cA1D	20.6 % ± 2 %	98.9 % ± 9.8 %	ANOVA with *post hoc* Bonferroni’s test
	cCD	23.6 % ± 1.1 %	112.9 % ± 5.2 %	
	c78	23 % ± 1.4 %	110.5 % ± 6.8 %	
	cA124BCD	23.1 % ± 1 %	110.8 % ± 5 %	
	coated control	20.9 % ± 1.4 %	100 % ± 6.8 %	
	sA1D	21.5 % ± 2.6 %	76.4 % ± 9.4 %	ANOVA with *post hoc*
	sCD	23.1 % ± 2.4 %	82.2 % ± 8.4 %	
	s78	28.7 % ± 2.6 %	102.1 % ± 9.2 %	

	sA124BCD	24.1 % ± 2.1 %	86 % ± 7.3 %	Bonferroni's test
	soluble control	28.1 % ± 3.1 %	100 % ± 10.9 %	
7 div	cA1D	42.1 % ± 2.2 %	119 % ± 6.3 %	Kruskal-Wallis test with *post hoc* Dunn's test
	cCD	38 % ± 2.5 %	107.2 % ± 7 %	
	c78	39.4 % ± 1.5 %	111.2 % ± 4.3 %	
	cA124BCD	33.3 % ±1.4 %	94.1 % ± 4 %	
	coated control	35.4 % ± 1.7 %	100 % ± 4.7 %	
	sA1D	37.4 % ± 2.7 %	112.1 % ± 8.1 %	Kruskal-Wallis test with *post hoc* Dunn's test
	sCD	40.8 % ± 3 %	122.3 % ± 8.9 %	
	s78	35.1 % ± 1.9 %	105.3 % ± 5.7 %	
	sA124BCD	41.6 % ± 3.3 %	125 % ± 10 %	
	soluble control	33.3 % ± 2.2 %	100 % ± 6.6 %	
	**O4**			
	**Domains**	**Value**	**Value normalized to control**	**Statistical test**
3 div	cA1D	2.4 % ± 0.4 %	94.6 % ± 15.8 %	Kruskal-Wallis test with *post hoc* Dunn's test
	cCD	2.5 % ± 0.3 %	95.2 % ± 11.7 %	
	c78	2.8 % ± 0.4 %	107.8 % ± 15 %	
	cA124BCD	2 % ± 0.4 %	78.6 % ± 14 %	
	coated control	2.6 % ± 0.4 %	100 % ± 16.7 %	
	sA1D	20.8 % ± 2.3 %	138.4 % ± 15.2 %	Kruskal-Wallis test with *post hoc* Dunn's test
	sCD	16.7 % ± 2.1 %	111 % ± 14.1 %	
	s78	15.1 % ± 1.6 %	100.9 % ± 10.5 %	
	sA124BCD	27.8 % ± 3.9 %	185.6 % ± 25.9 %	
	soluble control	15 % ± 2.8 %	100 % ± 18.4 %	
7 div	cA1D	5.2 % ± 0.8 %	139.7 % ± 20.6 %	ANOVA with *post hoc* Bonferroni's test
	cCD	5.6 % ± 0.6 %	149.6 % ± 16.2 %	
	c78	5.1 % ± 0.6 %	137.2 % ± 16.3 %	
	cA124BCD	4.6 % ± 0.7 %	124.9 % ± 18.8 %	
	coated control	3.7 % ± 0.8 %	100 % ± 20.2 %	
	sA1D	4.9 % ± 0.7 %	34.5 % ± 4.8	Kruskal-Wallis test with *post hoc* Dunn's test
	sCD	8.8 % ± 1.3 %	61.5 % ± 9.3 %	
	s78	4.7 % ± 0.9 %	32.8 % ±6.2 %	
	sA124BCD	20 % ± 2.8 %	139.6 % ± 19.7 %	
	soluble control	14.3 % ± 3.7 %	100 % ± 26 %	
	**Nestin**			
	**Domains**	**Value**	**Value normalized to control**	**Statistical test**
3 div	cA1D	55.4 % ± 1.6 %	94.6 % ± 2.8 %	Kruskal-Wallis test with *post hoc* Dunn's test
	cCD	58.7 % ± 2.7 %	100.2 % ± 4.5 %	
	c78	62.9 % ± 4.6 %	107.5 % ± 7.9 %	
	cA124BCD	71.4 % ± 7.6 %	122 % ± 13 %	
	coated control	58.5 % ± 1.7 %	100 % ± 2.8 %	
	sA1D	36.8 % ± 5.1 %	84.7 ± 11.8 %	Kruskal-Wallis test with *post hoc* Dunn's test
	sCD	43.2 % ± 4 %	99.6 % ± 9.2 %	
	s78	39.2 % ± 4.6 %	90.4 % ± 10.7 %	
	sA124BCD	37 % ± 5 %	85.3 % ± 11.6 %	
	soluble control	43.4 % ± 4.1 %	100 % ± 9.4 %	
7 div	cA1D	51.3 % ± 2.5 %	102.2 % ± 4.9 %	ANOVA with *post hoc* Bonferroni's test
	cCD	49.1 % ± 2.2 %	97.8 % ± 4.3 %	
	c78	51.3 % ± 1.9 %	102.2 % ± 3.8 %	
	cA124BCD	51.9 % ± 2.7 %	103.4 % ± 5.3 %	

	coated control	50.2 % ± 3 %	100 % ± 5.9 %	
	sA1D	48.8 % ± 2.4 %	133 % ± 6.5 %	Kruskal-Wallis test with *post hoc* Dunn's test
	sCD	48.9 % ± 2.9 %	133.3 % ± 8 %	
	s78	37 % ± 4.9 %	101 % ± 13.4 %	
	sA124BCD	37 % ± 3.7 %	100.8 % ± 10.1 %	
	soluble control	36.7 % ± 3.8 %	100 % ±10.4 %	
	**PH3**			
	**Domains**	**Value**	**Value normalized to control**	**Statistical test**
3 div	cA1D	8.3 % ± 0.7 %	134.2 % ± 10.7 %	Kruskal-Wallis test with *post hoc* Dunn's test
	cCD	6.4 % ± 0.7 %	103.6 % ± 11.6 %	
	c78	6.9 % ± 0.6 %	112.6 % ± 9.4 %	
	cA124BCD	6.5 % ± 0.6 %	105.7 % ± 10.4 %	
	coated control	6.2 % ± 0.7 %	100 % ± 10.9 %	
	sA1D	3.3 % ± 0.5 %	74.6 % ± 10.4 %	Kruskal-Wallis test with *post hoc* Dunn's test
	sCD	4.2 % ± 0.8 %	94.6 % ± 17.5 %	
	s78	5.3 % ± 0.6 %	119.4 % ± 13.8 %	
	sA124BCD	3.1 % ± 0.5 %	69.8 % ± 11.9 %	
	soluble control	4.5 % ± 0.8 %	100 % ± 17.8 %	
7 div	cA1D	3.8 % ± 0.6 %	140.9 % ± 21.3 %	ANOVA with *post hoc* Bonferroni's test
	cCD	2.8 % ± 0.4 %	101.3 % ± 13.2 %	
	c78	3 % ± 0.5 %	110.1 % ± 16.3 %	
	cA124BCD	2.6 % ± 0.4 %	96.8 % ± 14.9 %	
	coated control	2.7 % ± 0.4 %	100 % ± 13.8 %	
	sA1D	3.4 % ± 0.5 %	169.6 % ± 22.5 %	Kruskal-Wallis test with *post hoc* Dunn's test
	sCD	2.2 % ± 0.5 %	107.9 % ± 25 %	
	s78	2.1 % ± 0.3 %	102.4 % ± 12.9 %	
	sA124BCD	2.1 % ± 0.5 %	102.5 % ± 26.7 %	
	soluble control	2 % ± 0.5 %	100 % ± 26.3 %	

Supplementary Table 5: Collection of all evaluated and normalized values of the PCR

	Tubb3			
	**Domains**	**Value**	**Value normalized to control**	**Statistical test**
3 div	cA1D	0.42 ± 0.02	121.3 % ± 5 %	Kruskal-Wallis test with *post hoc* Dunn's test
	cCD	0.29 ± 0.05	84 % ± 15.3 %	
	c78	0.24 ± 0.04	68.1 % ± 12.1 %	
	cA124BCD	0.33 ± 0.02	95.6 % ± 6.6 %	
	coated control	0.35 ± 0.06	100 % ± 16.8 %	
	sA1D	0.39 ± 0.04	133.8 % ± 13.8 %	Kruskal-Wallis test with *post hoc* Dunn's test
	sCD	0.33 ± 0.05	112.4 % ± 17.9 %	
	s78	0.29 ± 0.02	99.8 % ± 7 %	
	sA124BCD	0.23 ± 0.03	78.3 % ± 10.6 %	
	soluble control	0.29 ± 0.03	100 % ± 9.1 %	
7 div	cA1D	0.69 ± 0.07	122.1 % ± 13.1 %	Kruskal-Wallis test with *post hoc* Dunn's test
	cCD	0.71 ± 0.05	125.1 % ± 9.3 %	
	c78	0.51 ± 0.04	90.8 % ± 6.6 %	
	cA124BCD	0.5 ± 0.24	88.3 % ± 42.3 %	
	coated control	0.57 ± 0.03	100 % ± 5.7 %	

	sA1D	0.5 ± 0.02	102.2 % ± 3.6 %	Kruskal-Wallis test with *post hoc* Dunn's test
	sCD	0.56 ± 0.08	115.6 % ± 15.8 %	
	s78	0.47 ± 0.07	96.1 ± 13.6 %	
	sA124BCD	0.4 ± 0.04	82.4 % ± 8.9 %	
	soluble control	0.49 ± 0.1	100 % ± 20.9 %	
	Gfap			
	**Domains**	**Value**	**Value normalized to control**	**Statistical test**
3 div	cA1D	0.66 ± 0.05	94.6 % ± 6.6 %	Kruskal-Wallis test with *post hoc* Dunn's test
	cCD	0.67 ± 0.16	95.7 % ± 22.9 %	
	c78	0.68 ± 0.14	98.1 % ± 20.6 %	
	cA124BCD	0.71 ± 0.13	102 % ± 18 %	
	coated control	0.7 ± 0.11	100 % 16.4 %	
	sA1D	0.31 ± 0.14	109.1 % ± 47.5 %	Kruskal-Wallis test with *post hoc* Dunn's test
	sCD	0.44 ± 0.09	152.4 % ± 33 %	
	s78	0.5 ± 0.08	174.8 % ± 28.7 %	
	sA124BCD	0.45 ± 0.09	157.9 % ± 31.5 %	
	soluble control	0.29 ± 0.12	100 % ± 41.3 %	
7 div	cA1D	0.78 ±0.13	108.2 % ± 17.9 %	Kruskal-Wallis test with *post hoc* Dunn's test
	cCD	0.8 ± 0.15	110.9 % ± 20.6 %	
	c78	0.69 ± 0.13	95.2 % ± 18.3 %	
	cA124BCD	1.15 ± 0.5	159.3 % ± 70.1 %	
	coated control	0.72 ± 0.04	100 % ± 6.1 %	
	sA1D	0.46 ± 0.12	82.7 % ± 21.1 %	Kruskal-Wallis test with *post hoc* Dunn's test
	sCD	0.61 ± 0.07	109.4 % ± 11.8 %	
	s78	0.59 ± 0.04	105.7 % ± 7.6 %	
	sA124BCD	0.69 ± 0.09	124.2 % ± 15.6 %	
	soluble control	0.56 ±0.05	100 % ± 8.9 %;	
	Pdgfra			
	**Domains**	**Value**	**Value normalized to control**	**Statistical test**
3 div	cA1D	0.79 ± 0.12	105 % ± 16.5 %	Kruskal-Wallis test with *post hoc* Dunn's test
	cCD	0.84 ± 0.12	111.7 % ± 16.2 %	
	c78	0.66 ± 0.1	87.9 % ± 13.6 %	
	cA124BCD	0.72 ± 0.11	96.5 % ± 15 %	
	coated control	0.75 ± 0.1	100 % ± 13.1 %	
	sA1D	1.05 ± 0.1	119 % ± 11.1 %	Kruskal-Wallis test with *post hoc* Dunn's test
	sCD	1.02 ± 0.09	116 % ± 9.9 %	
	s78	0.91 ± 0.05	102.7 % ± 5.7 %	
	sA124BCD	0.85 ± 0.04	96.6 % ± 4.7 %	
	soluble control	0.88 ± 0.02	100 % ± 2.7 %	
7 div	cA1D	0.81 ± 0.13	118.2 % ± 18.3 %	Kruskal-Wallis test with *post hoc* Dunn's test
	cCD	0.8 ± 0.14	116.6 % ± 20 %	
	c78	0.7 ± 0.09	102.1 % ± 13.8 %	
	cA124BCD	0.65 ± 0.2	94.2 % ± 29.4 %	
	coated control	0.68 ± 0.19	100 % ± 27.6 %	
	sA1D	0.9 ± 0.08	102.6 % ± 9.2 %	Kruskal-Wallis test with *post hoc* Dunn's test
	sCD	0.93 ± 0.07	105.2 % ± 8.1 %	
	s78	0.89 ± 0.07	101.6 % ± 8.1 %	
	sA124BCD	0.9 ± 0.03	102.3 % ± 3.6 %	
	soluble control	0.88 ± 0.04	100 % ± 4.3 %	
	Nes			

	Domains	Value	Value normalized to control	Statistical test
3 div	cA1D	0.49 ± 0.16	129.7 % ± 41.5 %	Kruskal-Wallis test with *post hoc* Dunn's test
	cCD	0.32 ± 0.08	84.3 % ± 21.2 %	
	c78	0.35 ± 0.05	92.5 % ± 13.5 %	
	cA124BCD	0.26 ± 0.04	68.2 % ± 9.4 %,	
	coated control	0.38 ± 0.1	100 % ± 27.6 %	
	sA1D	0.52 ± 0.04	141.5 % ± 9.8 %	Kruskal-Wallis test with *post hoc* Dunn's test
	sCD	0.39 ± 0.05	107.1 % ± 14.7 %	
	s78	0.45 ± 0.09	123.2 % ± 23.1 %	
	sA124BCD	0.21 ± 0.08	58.6 % ± 20.8 %	
	soluble control	0.37 ± 0.05	100 % ± 12.7 %	
7 div	cA1D	0.31 ± 0.05	76.9 % ± 11.4 %	Kruskal-Wallis test with *post hoc* Dunn's test
	cCD	0.31 ± 0.08	75.8 % ± 18.6 %	
	c78	0.32 ± 0.1	77.9 % ± 24.1 %	
	cA124BCD	0.58 ± 0.19	142.3 % ± 45.4 %	
	coated control	0.41 ± 0.13	100 % ± 32.7 %	
	sA1D	0.18 ± 0.03	88.6 % ± 15.1 %	Kruskal-Wallis test with *post hoc* Dunn's test
	sCD	0.26 ± 0.03	125.7 % ± 15.9 %	
	s78	0.24 ± 0.08	117.6 % ± 38.6 %	
	sA124BCD	0.09 ±0.003	44.1 % ± 1.5 %	
	soluble control	0.21 ± 0.09	100 % ± 44 %	
	Khdrbs1			
	**Domains**	**Value**	**Value normalized to control**	**Statistical test**
3 div	cA1D	0.22 ± 0.08	104.3 % ± 39.3 %	Kruskal-Wallis test with *post hoc* Dunn's test
	cCD	0.24 ± 0.09	115.6 % ± 43.3 %	
	c78	0.21 ± 0.07	100.4 % ± 32.2 %	
	cA124BCD	0.22 ± 0.08	104.5 % ± 37.5 %	
	coated control	0.21 ± 0.09	100 % ± 45.3 %	
	sA1D	0.66 ±0.12	132.7 % ± 23.6 %	Kruskal-Wallis test with *post hoc* Dunn's test
	sCD	0.68 ± 0.12	137.7 % ± 24 %	
	s78	0.52 ±0.09	105.5 % ± 17.8 %	
	sA124BCD	0.5 ± 0.04	100.2 % ± 8.9 %	
	soluble control	0.5 ±0.07	100 % ± 14 %	
7 div	cA1D	0.22 ± 0.1	92.6 % ± 43.1 %	Kruskal-Wallis test with *post hoc* Dunn's test
	cCD	0.23 ± 0.11	98.2 % ± 47.5 %	
	c78	0.2 ± 0.07	82.5 % ± 29.3 %	
	cA124BCD	0.27 ± 0.12	113.1 % ± 52 %	
	coated control	0.24 ± 0.13	100 % ± 54.2 %	
	sA1D	0.52 ± 0.03	123.1 % ± 6.2 %	Kruskal-Wallis test with *post hoc* Dunn's test
	sCD	0.5 ± 0.06	117.2 % ± 14.1 %	
	s78	0.52 ± 0.03	121.1 % ± 5.8 %	
	sA124BCD	0.6 ± 0.13	140.1 % ± 29.5 %	
	soluble control	0.43 ± 0.05	100 % ± 12.1 %	
	Vav3			
	**Domains**	**Value**	**Value normalized to control**	**Statistical test**
3 div	cA1D	0.08 ± 0.01	104.1 % ± 11.1 %	Kruskal-Wallis test with *post hoc* Dunn's test
	cCD	0.09 ± 0.02	120.7 % ± 23.3 %	
	c78	0.07 ± 0.01	89.3 % ± 6.5 %	
	cA124BCD	0.07 ± 0.01	96 % ± 8.5 %	

	coated control	0.07 ±0.01	100 % ± 17 %	
	sA1D	0.23 ± 0.07	119 % ± 34.6 %	Kruskal-Wallis test with *post hoc* Dunn's test
	sCD	0.26 ± 0.06	133.1 % ± 29.8 %	
	s78	0.22 ± 0.05	112.3 ± 26.1 %	
	sA124BCD	0.22 ± 0.04	115.1 % ± 21.7 %	
	soluble control	0.2 ± 0.04	100 % ± 20.5 %	
7 div	cA1D	0.08 ± 0.01	108.5 % ± 11.9 %	Kruskal-Wallis test with *post hoc* Dunn's test
	cCD	0.06 ± 0.004	77.4 % ± 5.9 %	
	c78	0.06 ± 0.01	84.3 % ± 13.4 %	
	cA124BCD	0.09 ± 0.02	118 % ± 21.1 %	
	coated control	0.08 ± 0.02	100 % ± 22.5 %	
	sA1D	0.17 ± 0.3	116.1 % ± 22.9 %	Kruskal-Wallis test with *post hoc* Dunn's test
	sCD	0.17 ± 0.02	119.5 % ± 15.1 %	
	s78	0.15 ± 0.02	102 % ± 14.6 %	
	sA124BCD	0.15 ± 0.04	103.6 % ± 26.6 %	
	soluble control	0.15 ± 0.07	100 % ± 45.2	
	Tnc			
	**Domains**	**Value**	**Value normalized to control**	**Statistical test**
3 div	cA1D	0.62 ± 0.16	118.4 % ± 30.9 %	Kruskal-Wallis test with *post hoc* Dunn's test
	cCD	0.5 ± 0.16	94.8 % ± 29.9 %	
	c78	0.49 ± 0.04	92.9 % ± 7.1 %	
	cA124BCD	0.62 ± 0.07	117.3 % ± 13 %	
	coated control	0.53 ± 0.06	100 % ± 10.7 %	
	sA1D	0.89 ± 0.11	108.2 % ± 13.3 %	Kruskal-Wallis test with *post hoc* Dunn's test
	sCD	0.97 ± 0.08	117.3 % ± 10.2 %	
	s78	0.92 ± 0.14	111.9 % ± 16.8 %	
	sA124BCD	0.83 ± 0.2	100.7 % ± 24 %	
	soluble control	0.82 ± 0.12	100 % ± 14 %	
7 div	cA1D	0.32 ± 0.12	98.9 % ± 36.5 %	Kruskal-Wallis test with *post hoc* Dunn's test
	cCD	0.32 ± 0.1	100.2 ± 30 %	
	c78	0.31 ± 0.1	95.4 % ± 30.9 %	
	cA124BCD	0.57 ± 0.21	175 % ± 65.4 %	
	coated control	0.32 ± 0.04	100 % ± 13 %	
	sA1D	0.52 ± 0.1	179 % ± 32.7 %	Kruskal-Wallis test with *post hoc* Dunn's test
	sCD	0.59 ± 0.11	200.6 % ± 38.9 %	
	s78	0.45 ± 0.11	155.4 % ± 36.6 %	
	sA124BCD	0.36 ± 0.09	123.2 % ± 29.9 %	
	soluble control	0.29 ± 0.09	100 % ± 29.9 %	

Supplementary Table 6: Collection of all evaluated and normalized values of the western blot analysis

	**Cleaved notch/notch**			
	**Domains**	**Value**	**Value normalized to control**	**Statistical test**
3 div	cA1D	0.55 ± 0.34	96.3 % ± 59.3 %	Kruskal-Wallis test with *post hoc* Dunn's test
	cCD	0.75 ± 0.47	130.2 % ± 82.4 %	
	c78	0.59 ± 0.33	101.8 % ± 57 %	
	cA124BCD	0.79 ± 0.45	137.2 % ± 77.8 %	
	coated control	0.58 ± 0.36	100 % ± 63 %	

	sA1D	0.48 ± 0.31	121.5 % ± 79.3 %	Kruskal-Wallis test with *post hoc* Dunn's test
	sCD	0.68 ± 0.41	174.4 % ± 105.6 %	
	s78	0.46 ± 0.25	116.9 % ± 65 %	
	sA124BCD	0.43 ± 0.28	108.9 % ± 72.8 %	
	soluble control	0.39 ±0.26	100 % ± 67.1 %	
7 div	cA1D	0.34 ± 0.21	89.7 % ± 53.6 %	Kruskal-Wallis test with *post hoc* Dunn's test
	cCD	0.69 ± 0.5	180.4 % ± 131.3 %	
	c78	0.62 ± 0.29	162.4 % ± 76.9 %	
	cA124BCD	0.86 ± 0.39	224.3 % ± 101.5 %	
	coated control	0.38 ± 0.2	100 % ± 52.4 %	
	sA1D	0.33 ± 0.19	72.4 % ± 41.9 %	Kruskal-Wallis test with *post hoc* Dunn's test
	sCD	0.51 ± 0.31	111.4 % ± 68.3 %	
	s78	0.43 ± 0.25	93.8 % ± 55.1 %	
	sA124BCD	0.46 ± 0.26	100.9 % ± 56 %	
	soluble control	0.46 ± 0.36	100 % ± 78.2 %	
	**pErk/tErk**			
	**Domains**	**Value**	**Value normalized to control**	**Statistical test**
3 div	cA1D	4.34 ± 0.81	149 % ± 27.6 %	Kruskal-Wallis test with *post hoc* Dunn's test
	cCD	4.49 ± 0.61	154.1 % ± 20.9 %	
	c78	3.12 ± 0.3	107.1 % ± 10.4 %	
	cA124BCD	3.87 ± 0.46	132.7 % ± 15.8 %	
	coated control	2.91 ± 0.51	100 % ± 17.5 %	
	sA1D	5.7 ± 0.5	67.5 % ± 5.9 %	Kruskal-Wallis test with *post hoc* Dunn's test
	sCD	5.29 ± 0.84	62.8 % ± 10 %	
	s78	6.39 ± 1.16	75.8 % ± 13.7 %	
	sA124BCD	7.48 ± 0.49	88.7 % ± 5.8 %	
	soluble control	8.43 ± 1.19	100 % ± 14.1 %	
7 div	cA1D	5.37 ± 2.42	153.2 % ± 69 %	Kruskal-Wallis test with *post hoc* Dunn's test
	cCD	4.16 ± 0.79	118.7 % ± 22.5 %	
	c78	4.18 ± 0.82	119.2 % ± 23.4 %	
	cA124BCD	3.88 ± 0.89	110.7 % ± 25.4 %	
	coated control	3.51 ± 0.29	100 % ± 8.4 %	
	sA1D	4.39 ± 0.28	65.1 % ± 4.1 %	Kruskal-Wallis test with *post hoc* Dunn's test
	sCD	4.75 ± 0.82	70.5 % ± 12.1 %	
	s78	5.08 ± 0.34	75.4 % ± 5.1 %	
	sA124BCD	5.22 ± 0.07	77.5 % ± 1.1 %	
	soluble control	6.74 ± 0.76	100 % ± 11.2 %	
	**pAkt/tAkt**			
	**Domains**	**Value**	**Value normalized to control**	**Statistical test**
3 div	cA1D	6.15 ± 1.89	107 % ± 33 %	Kruskal-Wallis test with *post hoc* Dunn's test
	cCD	6.09 ± 1.68	105.9 % ± 29.2 %	
	c78	6.15 ± 1.55	107 % ± 27 %	
	cA124BCD	5.96 ± 1.62	103.7 % ± 28.2 %	
	coated control	5.75 ±1.81	100 % ± 31.5 %	
	sA1D	3.05 ± 0.6	148.4 % ± 28.2 %	Kruskal-Wallis test with *post hoc* Dunn's test
	sCD	4.19 ± 0.94	203.6 % ± 45.8 %	
	s78	2.56 ± 0.57	124.5 % ± 27.6 %	
	sA124BCD	3.37 ± 0.95	163.5 % ± 46.2 %	
	soluble control	2.06 ± 0.48	100 % ± 23.5 %	

	Domains	Value	Value normalized to control	Statistical test
7 div	cA1D	6.62 ± 2.2	125.9 % ± 41.9 %	Kruskal-Wallis test with *post hoc* Dunn's test
	cCD	5.4 ± 1.21	102.6 % ± 23.1 %	
	c78	6.63 ± 2.1	126.1 % ± 40 %	
	cA124BCD	6.88 ± 2.45	130.8 ± 46.6 %	
	coated control	5.26 ± 1.97	100 % ± 37.5 %	
	sA1D	4.21 ±1.14	107.6 % ± 29 %	Kruskal-Wallis test with *post hoc* Dunn's test
	sCD	5.18 ± 1.51	132.3 % ± 38.5 %	
	s78	4.89 ± 1.45	125 % ± 37.1 %	
	sA124BCD	5.41 ± 1.64	138.2 % ± 41.8 %	
	soluble control	3.92 ± 1.43	100 % ± 36.6 %	
	**pFAK/tFAK**			
	**Domains**	**Value**	**Value normalized to control**	**Statistical test**
3 div	cA1D	0.23 ± 0.09	93.7 % ± 34.7 %	Kruskal-Wallis test with *post hoc* Dunn's test
	cCD	0.39 ± 0.24	159.4 % ± 98.2 %	
	c78	0.25 ± 0.08	100.3 % ± 31.2 %	
	cA124BCD	0.21 ± 0.05	86.7 % ± 18.8 %	
	coated control	0.25 ± 0.05	100 % ±19.4 %	
	sA1D	0.13 ± 0.05	115.5 % ± 46.3 %	Kruskal-Wallis test with *post hoc* Dunn's test
	sCD	0.1 ± 0.002	90.1 % ± 2.1 %	
	s78	0.14 ± 0.03	117.5 % ± 22.6 %	
	sA124BCD	0.13 ± 0.01	112 % ± 6.5 %	
	soluble control	0.12 ± 0.03	100 % ± 23.2 %	
7 div	cA1D	0.19 ± 0.05	89.8 % ± 21.1 %	Kruskal-Wallis test with *post hoc* Dunn's test
	cCD	0.18 ± 0.03	84 % ± 12.4 %	
	c78	0.23 ± 0.03	105.8 % ± 15.2 %	
	cA124BCD	0.27 ± 0.08	129.6 % ± 38.8 %	
	coated control	0.21 ± 0.04	100 % ± 16.7 %	
	sA1D	0.14 ± 0.02	87.3 % ± 10.6 %	Kruskal-Wallis test with *post hoc* Dunn's test
	sCD	0.16 ± 0.03	98.6 % ± 20.7 %	
	s78	0.15 ± 0.02	91.8 % ± 15.2 %	
	sA124BCD	0.16 ± 0.03	94.9 % ± 17.4 %	
	soluble control	0.16 ± 0.02	100 % ± 13.7 %	
	**Sam68/α-tubulin**			
	**Domains**	**Value**	**Value normalized to control**	**Statistical test**
3 div	cA1D	1.24 ± 0.76	205.5 % ± 125.8 %	Kruskal-Wallis test with *post hoc* Dunn's test
	cCD	1.51 ± 0.76	251 % ± 126.8 %	
	c78	1.6 ± 0.82	265.7 % ± 136.3 %	
	cA124BCD	1.75 ± 0.87	290.2 % ±144.2 %	
	coated control	0.6 ± 0.53	100 % ± 88 %	
	sA1D	1.9 ± 0.94	151 % ± 74.5 %	Kruskal-Wallis test with *post hoc* Dunn's test
	sCD	2.48 ± 1.15	197.2 % 91.9 %	
	s78	1.7 ± 0.64	135.3 % ± 51.1 %	
	sA124BCD	1.87 ± 0.55	148.7 % ± 44.1 %	
	soluble control	1.26 ± 0.45	100 % ± 35.6 %	
7 div	cA1D	0.72 ± 0.32	139 % ± 62 %	Kruskal-Wallis test with *post hoc* Dunn's test
	cCD	0.3 ± 0.12	58.1 % ± 22.3 %	
	c78	0.19 ± 0.09	37.4 % ± 17.9 %	
	cA124BCD	0.5 ± 0.26	96.4 % ± 49.4 %	
	coated control	0.52 ± 0.3	100 % ± 56 %	
	sA1D	0.94 ± 0.51	229.7 % ± 125.8 %	

	sCD	0.65 ± 0.4	160.1 % ± 98.6 %	Kruskal-Wallis test with *post hoc* Dunn's test
	s78	1.1 ± 0.6	269.1 % ± 150.5 %	
	sA124BCD	0.82 ± 0.62	202.6 % ± 152.8 %	
	soluble control	0.41 ± 0.2	100 % ± 49.1 %	

Supplementary Table 7: Collection of all evaluated and normalized values of the video microscopy

Distance per day				
	**Domains**	**Value**	**Value normalized to control**	**Statistical test**
4 div	cA1D	273.1 µm/d ± 18.2 µm/d	108.9 % ± 7.2 %	Kruskal-Wallis test with *post hoc* Dunn's test
	cCD	305.8 µm/d ± 21 µm/d	121.9 % ± 8.4 %	
	c78	408 µm/d ± 26 µm/d	162.6 % ± 10.4 %	
	cA124BCD	259.3 µm/d ± 17.7 µm/d	103.3 % ± 7.1 %	
	coated control	250.9 µm/d ± 18.6 µm/d	100 % ± 7.4 %	
	sA1D	391.6 µm/d ±27.7 µm/d	77.5 % ± 5.5 %	Kruskal-Wallis test with *post hoc* Dunn's test
	sCD	384.6 µm/d ± 27 µm/d	76.1 % ± 5.4 %	
	s78	535.7 µm/d ± 27.7 µm/d	106 % ± 5.5 %	
	sA124BCD	279.3 µm/d ± 14.2 µm/d	55.2 % ± 2.8 %	
	soluble control	505.6 µm/d ± 27.5 µm/d	100 % ± 5.5 %	
	**Distance per hour**			
	**Domains**	**Value**	**Value normalized to control**	**Statistical test**
4 div	cA1D	11.4 µm/h ± 0.8 µm/h	108.8 % ± 7.2 %	Kruskal-Wallis test with *post hoc* Dunn's test
	cCD	12.7 µm/h ± 0.9 µm/h	121.8 % ± 8.4 %	
	c78	17 µm/h ± 1.1 µm/h	162.5 % ± 10.4 %	
	cA124BCD	10.8 µm/h ± 0.7 µm/h	103.3 % ± 7.1 %	
	coated control	10.5 µm/h ± 0.8 µm/h	100 % ± 7.4 %	
	sA1D	16.3 µm/h ± 1.2 µm/h	77.5 % ± 5.5 %	Kruskal-Wallis test with *post hoc* Dunn's test
	sCD	16 µm/h ± 1.1 µm/h	76.1 % ± 5.4 %	
	s78	22.3 µm/h ±1.2 µm/h	105.9 % ± 5.5 %	
	sA124BCD	11.6 µm/h ± 0.6 µm/h	55.2 % ± 2.8 %	
	soluble control	21.1 µm/h ± 1.2 µm/h	100 % ± 5.5 %	
	**Average of cell divisions**			
	**Domains**	**Value**	**Value normalized to control**	**Statistical test**
4 div	cA1D	0.28 ± 0.05	69.7 % ± 11.3 %	Kruskal-Wallis test with *post hoc* Dunn's test
	cCD	0.24 ± 0.04	59 % ± 9.5 %	
	c78	0.38 ± 0.05	94.4 % ± 12.7 %	
	cA124BCD	0.23 ± 0.04	56.7 % ± 9.4 %	
	coated control	0.4 ± 0.06	100 % ± 14.5 %	
	sA1D	0.57 ±0.06	80.2 % ± 8.7 %	
	sCD	0.71 ± 0.11	100.5 % ± 15.1 %	

	s78	0.91 ± 0.14	129.3 % ± 20 %	Kruskal-Wallis test with *post hoc* Dunn's test
	sA124BCD	0.42 ± 0.06	59.2 % ± 8.6 %	
	soluble control	0.71 ± 0.07	100 % ± 9.9 %	
